# Supplementary material for: Liver TET1 promotes metabolic dysfunction-associated steatotic liver disease
Source: EMBO Mol Med. 2025 Mar 31;17(5):1101–17. doi: 10.1038/s44321-025-00224-4 (PMC12081649; doi:10.1038/s44321-025-00224-4)
Supplement: Supplementary file 2 — Appendix [file 44321_2025_224_MOESM2_ESM.pdf]

Appendix

**Table of content**	**Page numbers**
**Appendix Figure S1. The mRNA expression levels of DNMTs in MAFLDs.**	**2**
**Appendix Figure S2. The mRNA expression levels of the TET family enzymes in MAFLDs.**	**3**
**Appendix Figure S3. TET1 deficiency has no significant impact on mouse liver development.**	**4**
**Appendix Figure S4. The impacts of TET1 deficiency on mouse food, water consumption, and fed glucose change.**	**5**
**Appendix Figure S5. Differentially expressed hepatokines between control and TET1 KO mice.**	**6**
**Appendix Figure S6. Lipidomic analysis of control, TET1 LKO, and TET1 KO mice.**	**7**
**Appendix Figure S7. Characterization of liver specific TET1 knockout mice.**	**8**
**Appendix Figure S8. Metabolic changes in the liver specific TET1 KO mice.**	**9**
**Appendix Figure S9. The impact of TET1 on Pparα and Hnf4α signaling cascade.**	**10**

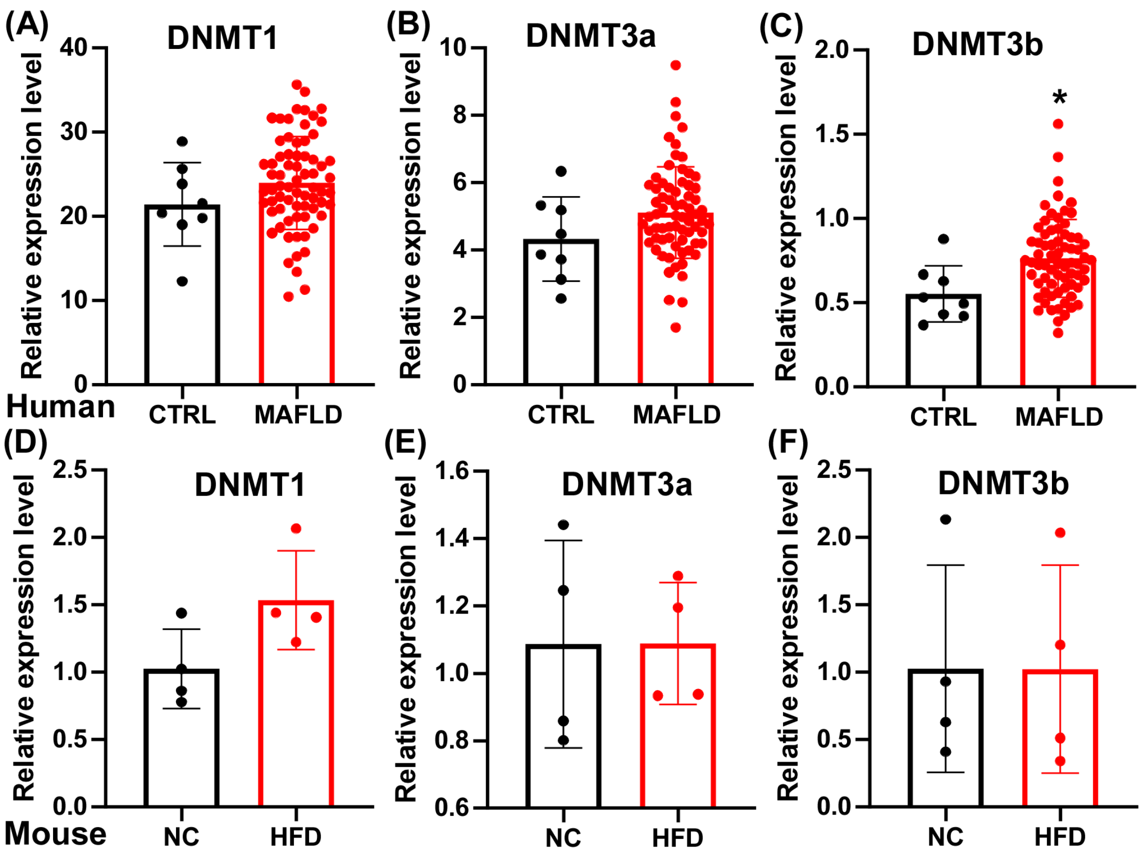

Appendix Figure S1. The mRNA expression levels of DNMTs in MAFLDs. (A) DNMT1, (B) DNMT3a, and (C) DNMT3b were examined in the liver samples derived from control (CTRL) and MAFLD patients, n=8 in CTRL and n=70 in MAFLD. The human data was retrieved from GSE174478 dataset. Similarly, DNMT1, DNMT3a, and DNMT3b were determined in the liver samples derived from male mice fed with normal chew (NC) or high fat diet (HFD) for 16 weeks, n=4. The mouse data was retrieved from GSE57425 dataset. *p<0.05, compared with the control group.

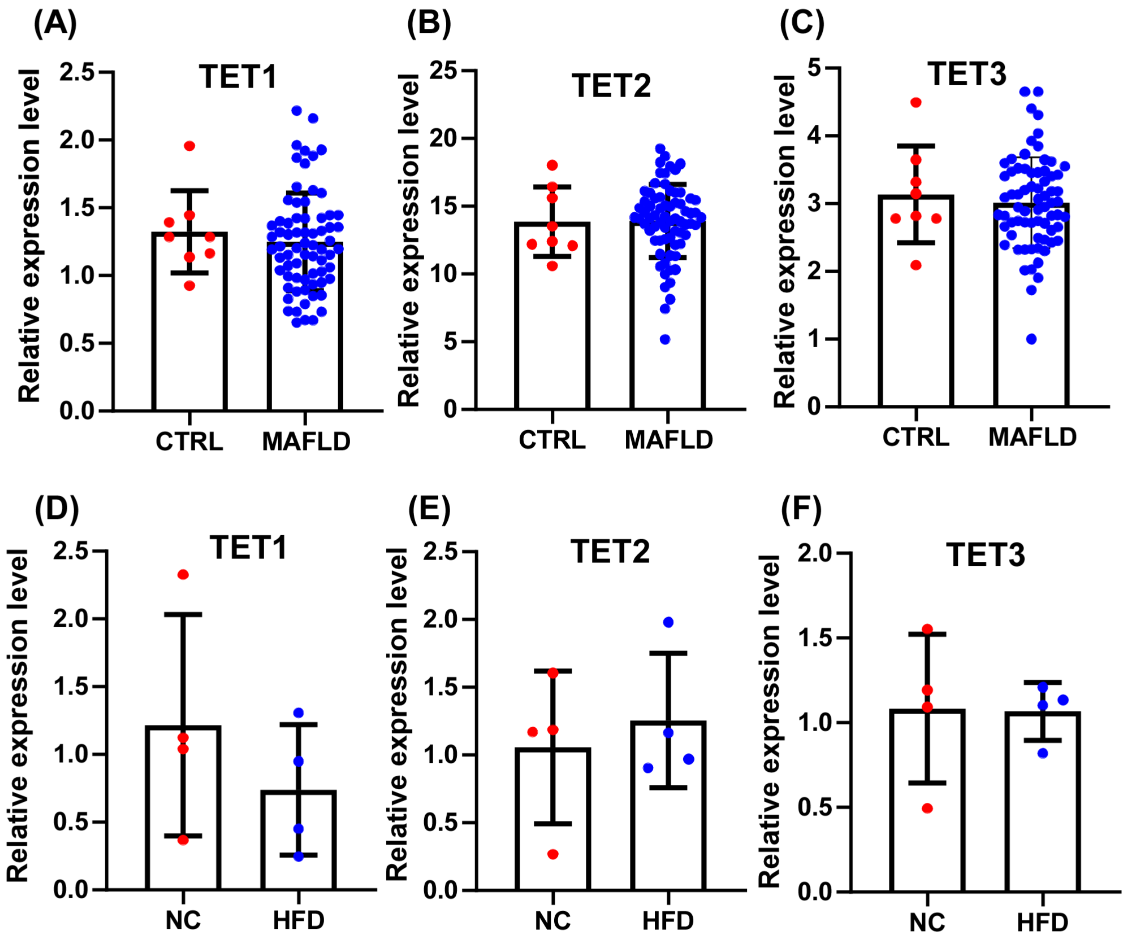

Appendix Figure S2. The mRNA expression levels of the TET family enzymes in MAFLDs. (A) TET1, (B) TET2, and (C) TET3 mRNA expression levels were examined in the human liver samples derived from CTRL and MAFLD patients, n=8 in CTRL and n=70 in MAFLD (GSE174478). Similarly, (D) TET1, (E) TET2, and (F) TET3 were determined in the liver samples of NC and HFD fed male mice, n=4 (GSE57425).

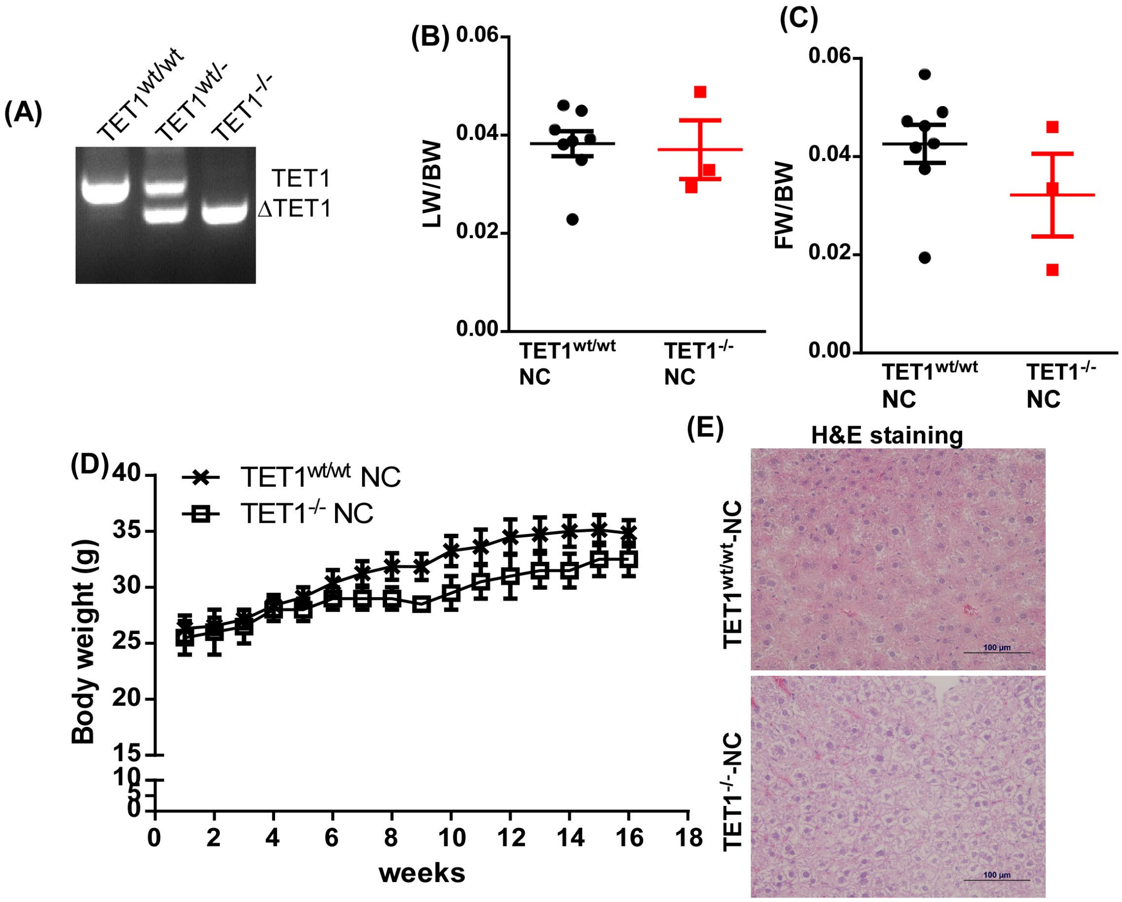

Appendix Figure S3.. TET1 deficiency has no significant impact on mouse liver development. (A) genotyping (Forward primer: GCTTCTCAGACTAGTGCTCTTC, reverse primer: AGAACCATCCAACTCACACTC), ΔTET1 indicates TET1 deletion (B) LW/BW and (C) FW/BW were quantified in the control and TET1 KO male mice fed with an NC. (D) Body weight of these male mice were measured post-NC feeding starting from 8-weeks. (E) H&E staining results were examined in these mice.

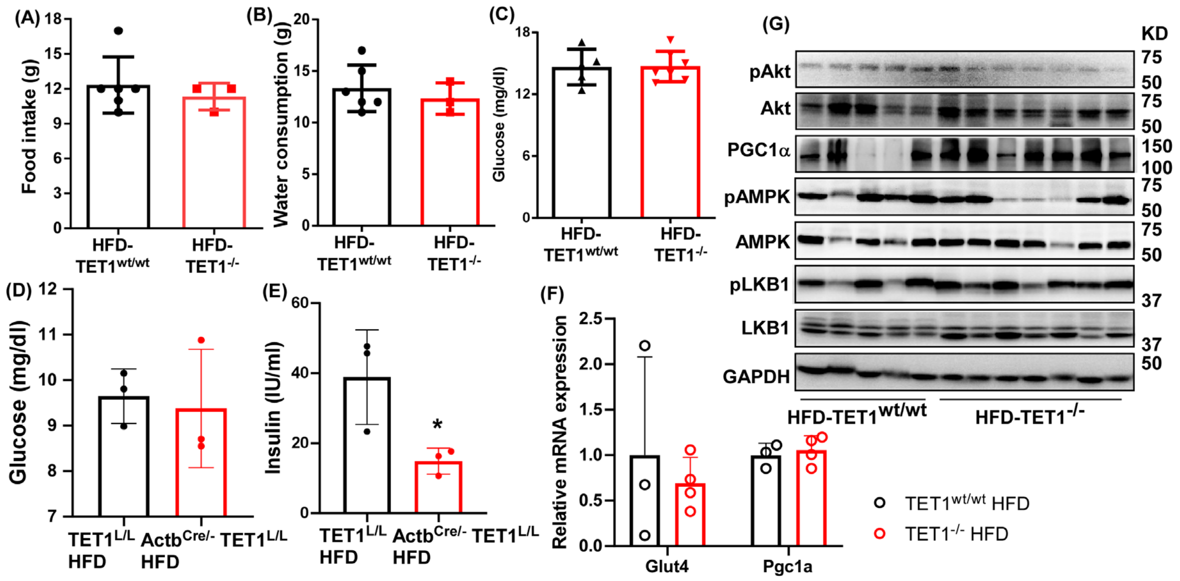

Appendix Figure S4. The impacts of TET1 deficiency on mouse food, water consumption, and fed glucose change. (A) Food weight and (B) water volume were measured during a 4-days feeding period in male control and TET1 KO mice fed with HFD for 12 weeks. (C) Glucose were measured in male TET1$^{wt/wt}$ and TET1$^{-/-}$ mice fed with an HFD. (D) Glucose and (E) insulin levels were measured in male Actb$^{Cre/-}$ TET1$^{L/L}$ and TET1$^{L/L}$ mice fed with an HFD for 16 weeks. (F) GLUT4 and PGC1a mRNA expression levels were examined in the liver samples of male TET1$^{wt/wt}$ and TET1$^{-/-}$ mice. (F) Akt, phosphorylated Akt (p-Akt), PGC1a, and GADPH were determined in the livers derived from the male TET1$^{wt/wt}$ and TET1$^{-/-}$ mice as indicated. *, $p<0.05$; when compared with the relevant control group.

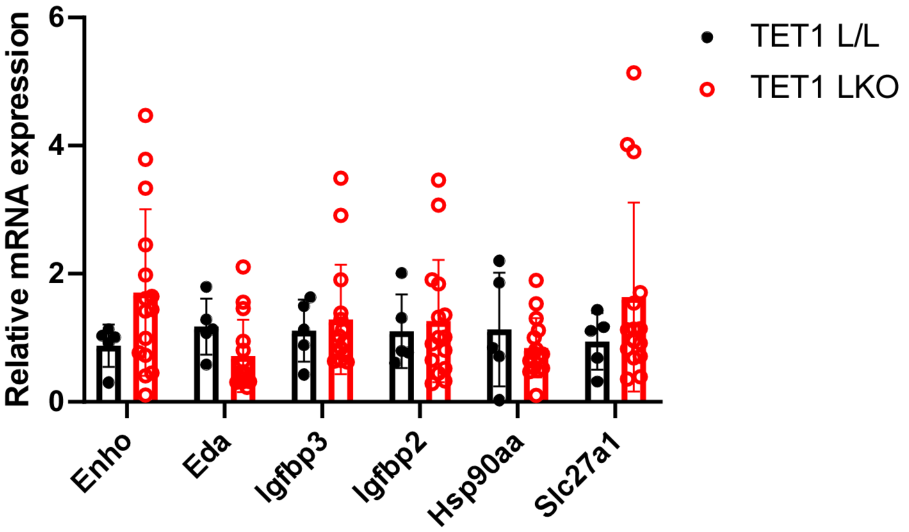

Appendix Figure S5.. Differentially expressed hepatokines between control and TET1 KO mice. The identified significantly altered Hepatokines including Enho, Eda, Igfbp2, Igfbp3, Hsp90aa, and Slc27a1 in TET1 KO mice were examined in TET1 LKO mice.

(A)

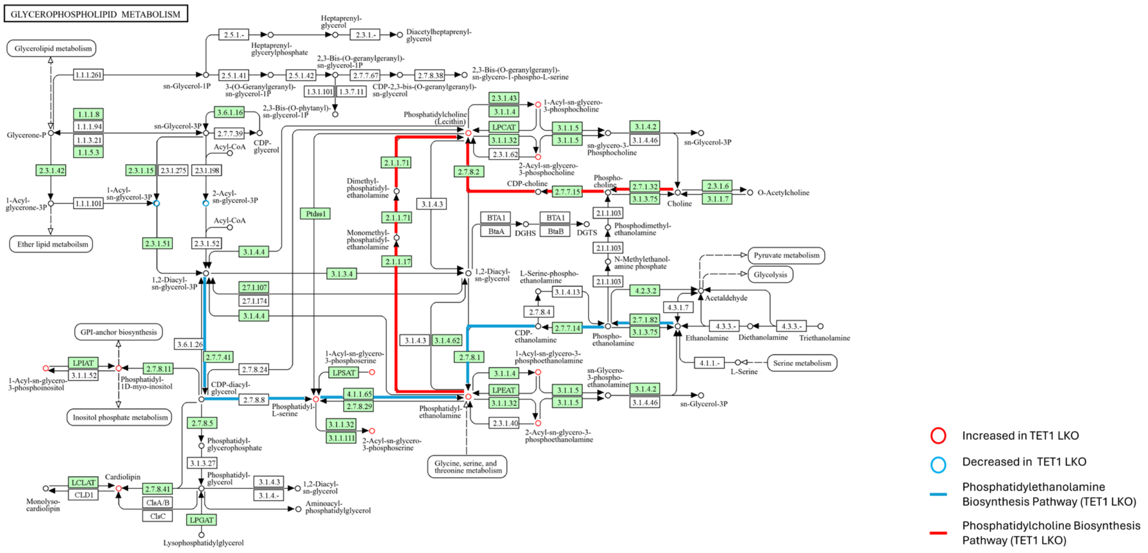

(B)

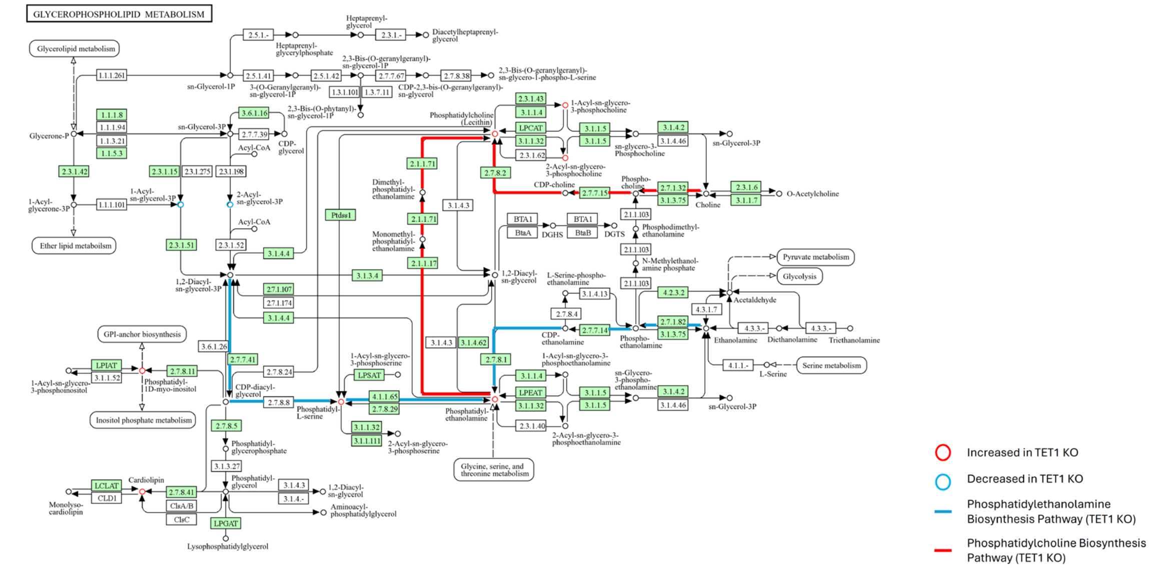

Appendix Figure S6. Lipidomic analysis of control, TET1 LKO, and TET1 KO mice. The most significantly altered lipids and their relevant pathways in TET1 KO and TET1 LKO mice were displayed. (A) The altered lipid pathways in TET1 LKO mice versus the control ones. (B) The altered lipid pathways in TET1 KO mice versus the control ones.

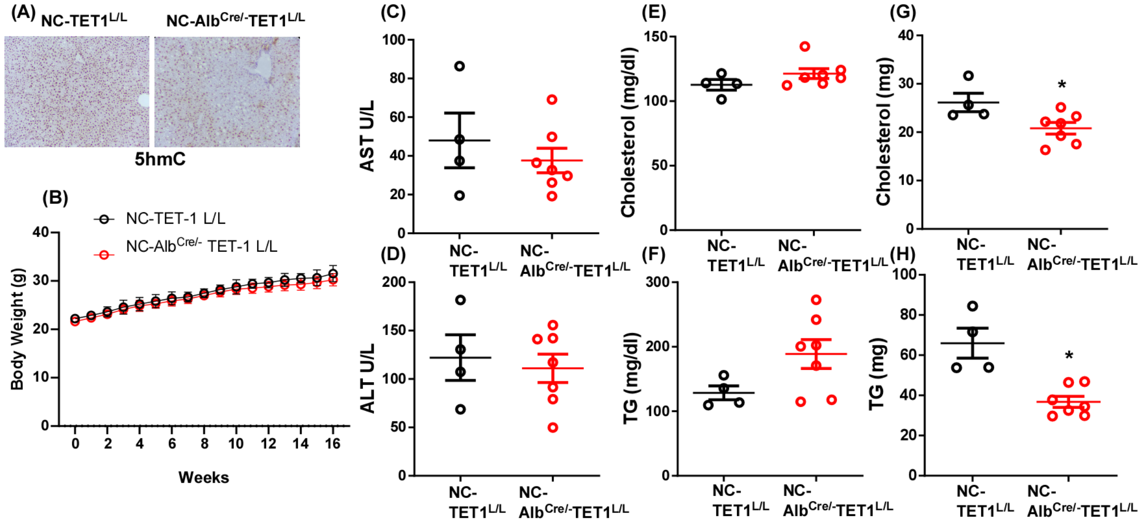

Appendix Figure S7. Characterization of liver specific TET1 knockout mice. (A) IHC staining of 5hmC in the liver samples derived from male WT and liver specific TET1 knockout (TET1 LKO) mice. (B) BW of WT and TET1 LKO mice were measured weekly post-NC treatment starting from 8-weeks. (C) AST, (D) ALT, (E) serum cholesterols, (F) serum triglycerides (TG) (G) hepatic cholesterols, and (H) hepatic TG were determined in WT and TET1 LKO mice fed with an NC diet for 16 weeks. *, $p<0.05$, when compared with the relevant control group.

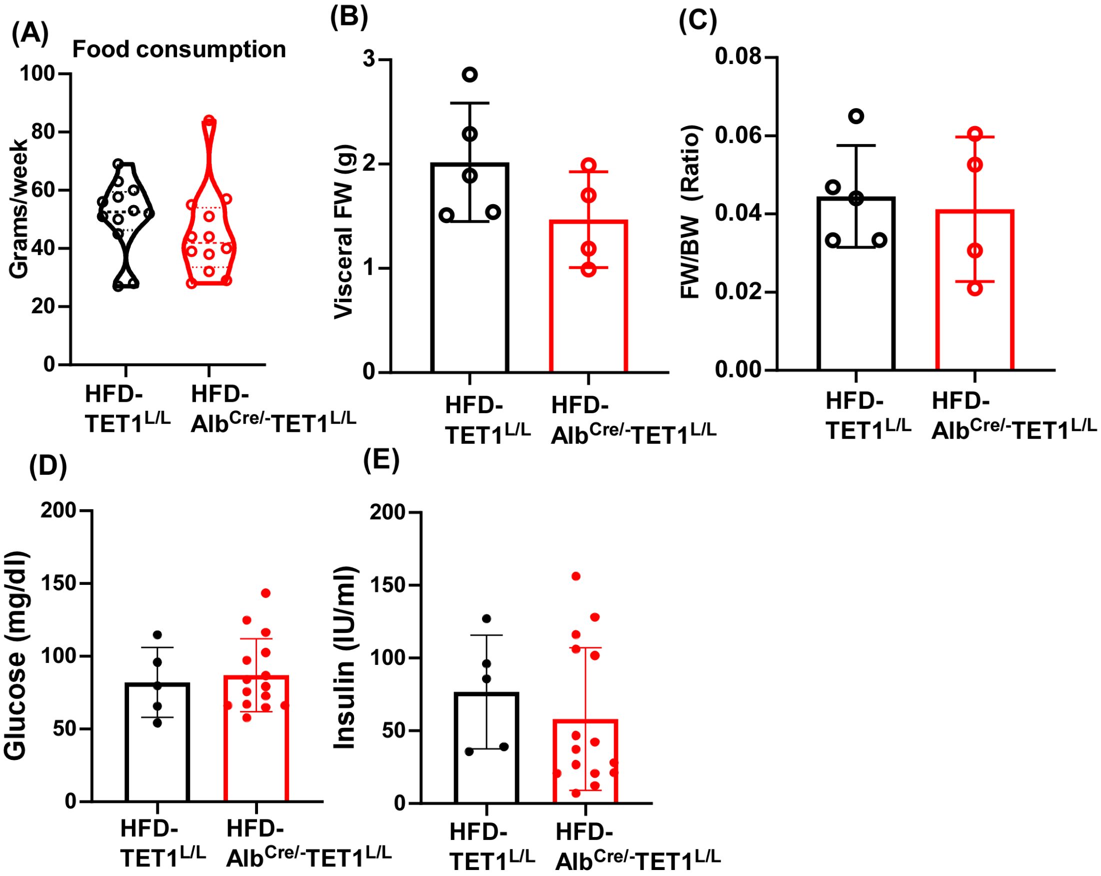

Appendix Figure S8. Metabolic changes in the liver specific TET1 KO mice. (A) Food consumption was determined in male control and TET1 LKO mice fed with an HFD. (B) FW, (C) FW/BW, (D) fed glucose, and (E) fed insulin were measured in male control and TET1 LKO mice fed with an HFD for 16 weeks.

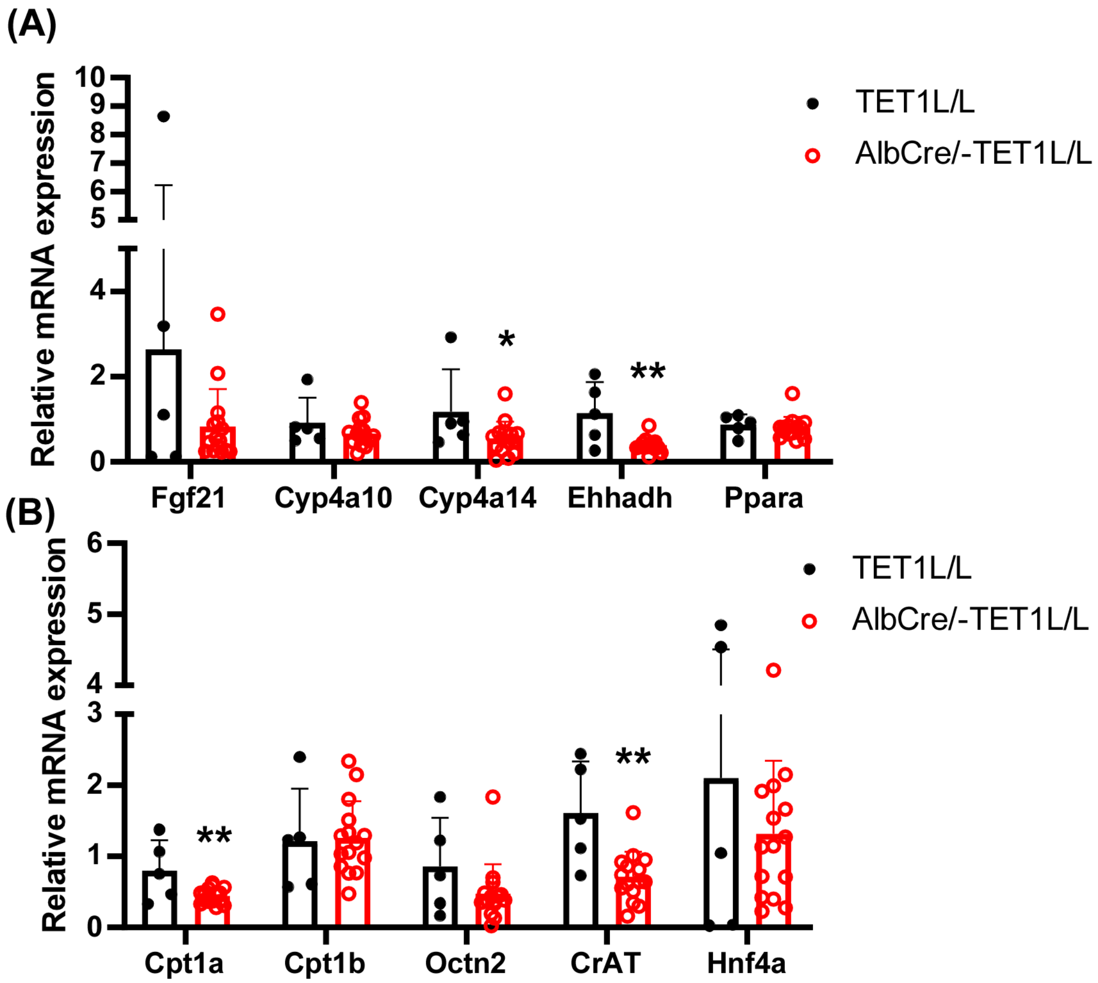

Appendix Figure S9. The impact of TET1 on Pparα and Hnf4α signaling cascade. The signaling cascades of Pparα and Hnf4α which sensor free fatty acid were downregulated in liver specific TET1 knockout mice. (A) The mRNA expression levels of Fgf21, Cyp4a10, Cyp4a14, Ehhadh, and Ppara were examined in male control and TET1 LKO mice fed with an HFD for 16 weeks. (B) The mRNA expression levels of Cpt1a, Cpt1b, Octn2, CrAT, and Hnf4a were determined in these mice. *, $p<0.05$; **, $p<0.01$; when compared with the relevant control group.
