# Supplementary material for: Liver TET1 promotes metabolic dysfunction-associated steatotic liver disease
Source: EMBO Mol Med. 2025 Mar 31;17(5):1101–17. doi: 10.1038/s44321-025-00224-4 (PMC12081649; doi:10.1038/s44321-025-00224-4)
Supplement: Supplementary file 8 — Data Set [file 44321_2025_224_MOESM8_ESM.zip › Readme.docx]

Dataset EV1: RNA sequencing data from liver samples of wild-type (WT; n = 3) and whole-body Tet1 knockout (n = 4) mice fed a high-fat diet (HFD).

Dataset EV2: Differentially expressed hepatokines identified from RNA sequencing data, highlighting the most significantly regulated genes.

Dataset EV3: Comparative lipidomic profiling of liver samples from wild-type (G1; n = 5) and liver-specific Tet1 knockout (G2; n=5) mice fed a high-fat diet (HFD).

Dataset EV4: Comparative lipidomic profiling of liver samples from wild-type (G1; n = 5) and whole-body Tet1 knockout mice (G3; n=3) fed a high-fat diet (HFD).

Table EV1: Experimental group arrangements and sample details corresponding to Datasets EV3 and EV4.
