# Supplementary figures and images for: Liver TET1 promotes metabolic dysfunction-associated steatotic liver disease
Source: EMBO Mol Med. 2025 Mar 31;17(5):1101–17. doi: 10.1038/s44321-025-00224-4 (PMC12081649; doi:10.1038/s44321-025-00224-4)

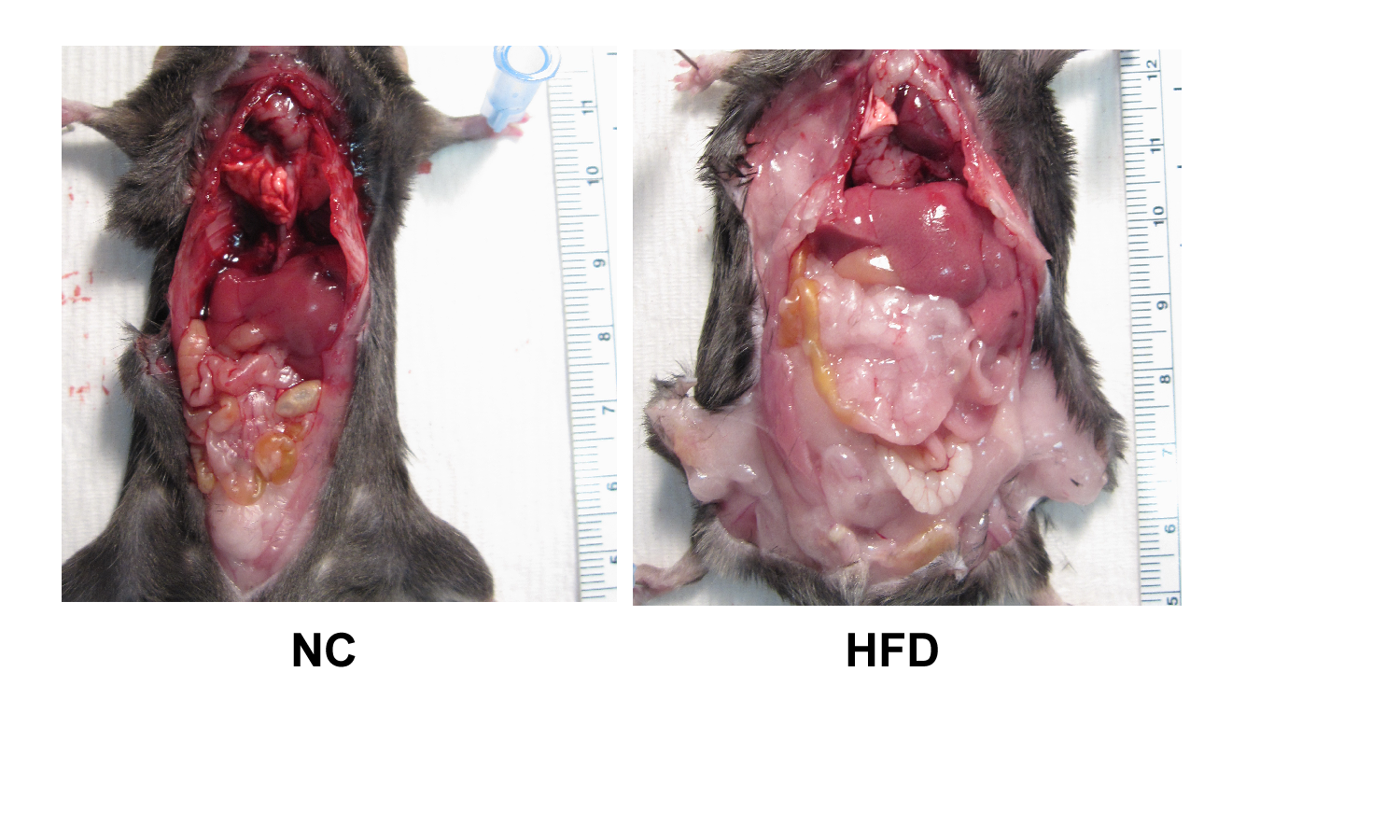

Supplement: Supplementary file 9 — Source data Fig. 1 [file 44321_2025_224_MOESM9_ESM.zip › Fig 1/Fig 1B.tif]

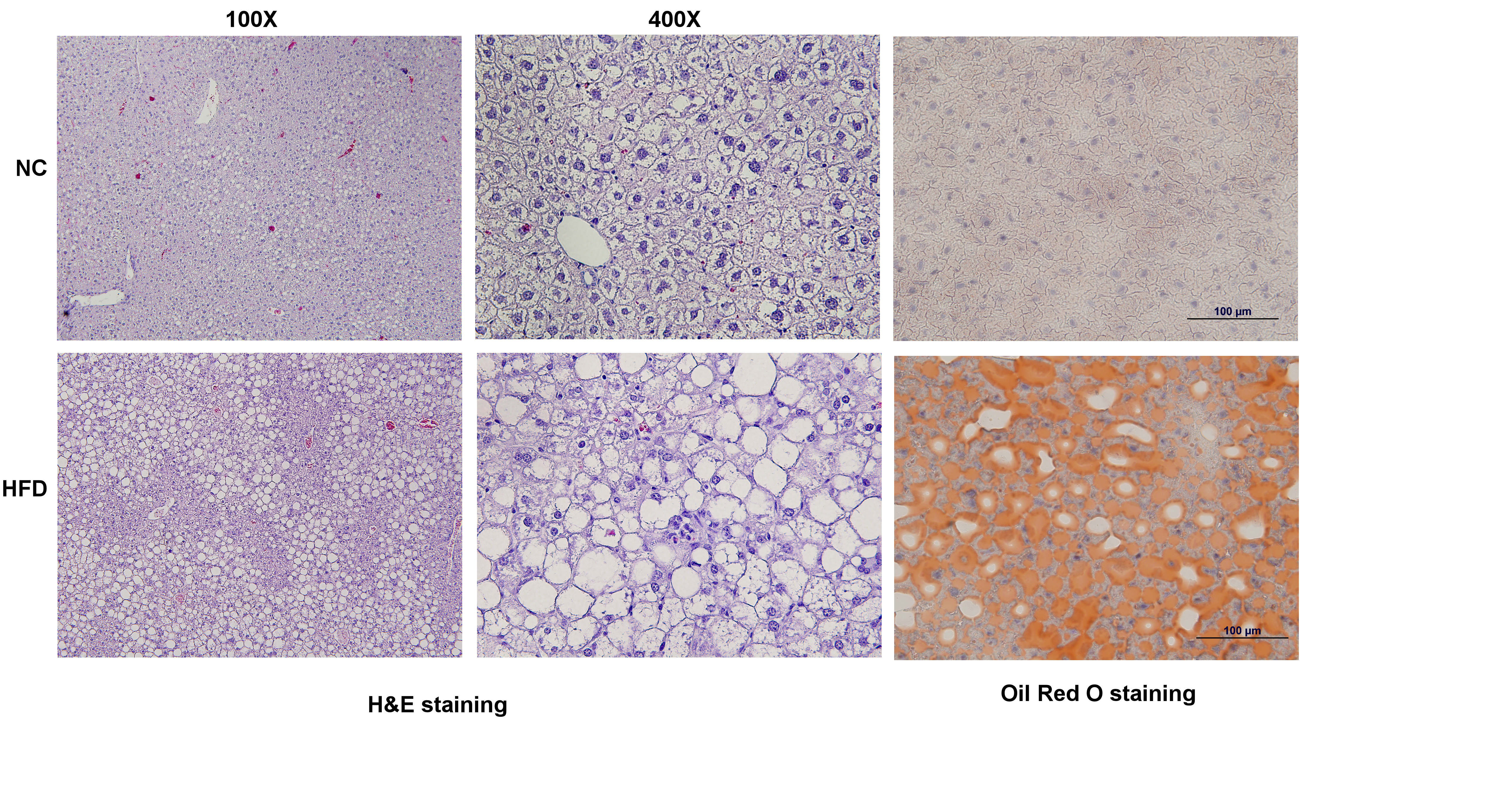

Supplement: Supplementary file 9 — Source data Fig. 1 [file 44321_2025_224_MOESM9_ESM.zip › Fig 1/Fig 1C.tif]

## Slide 1
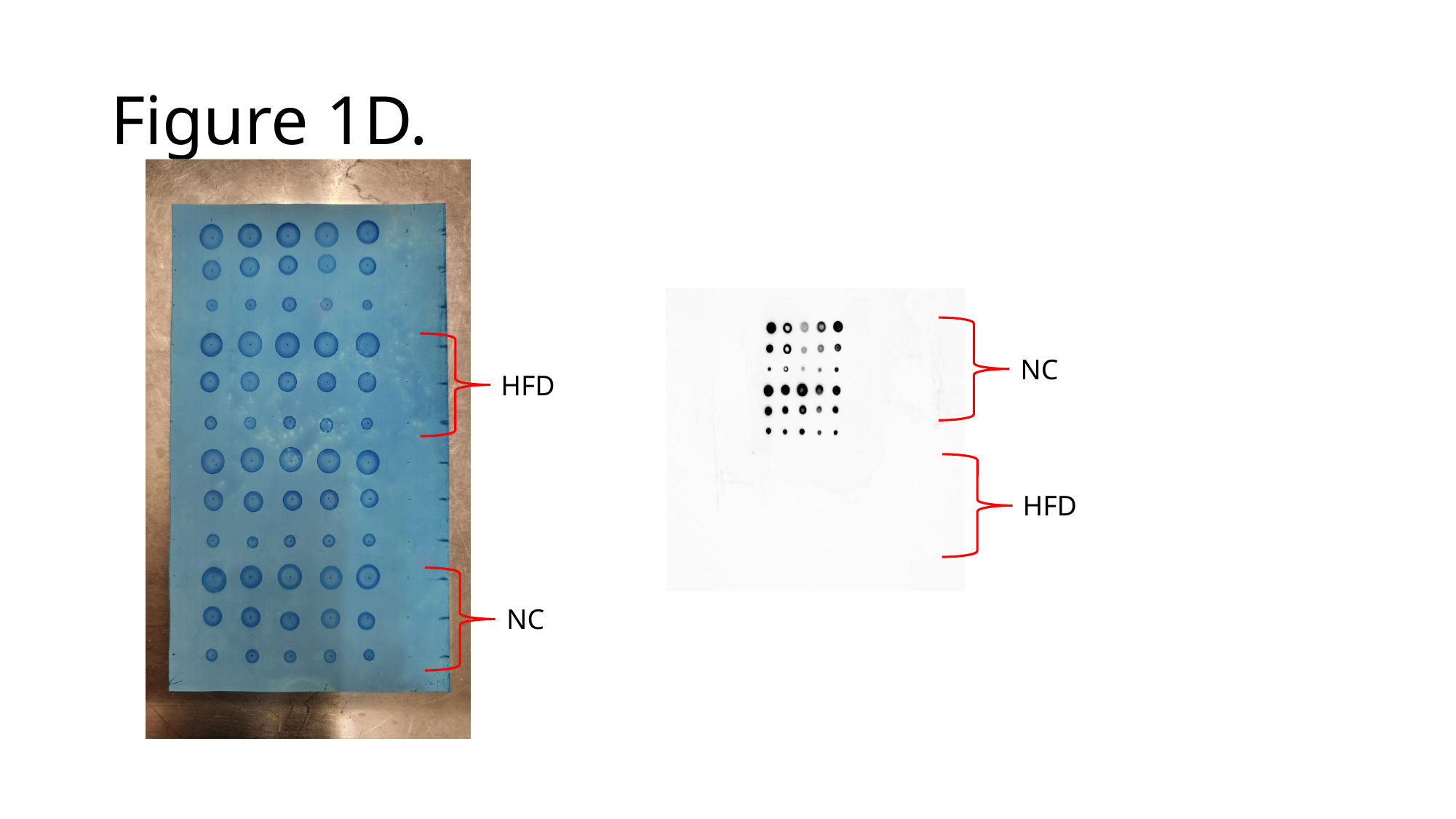

# Figure 1D.
NC
HFD
HFD
NC

Supplement: Supplementary file 9 — Source data Fig. 1 [file 44321_2025_224_MOESM9_ESM.zip › Fig 1/Fig 1D.pptx]

## Slide 1
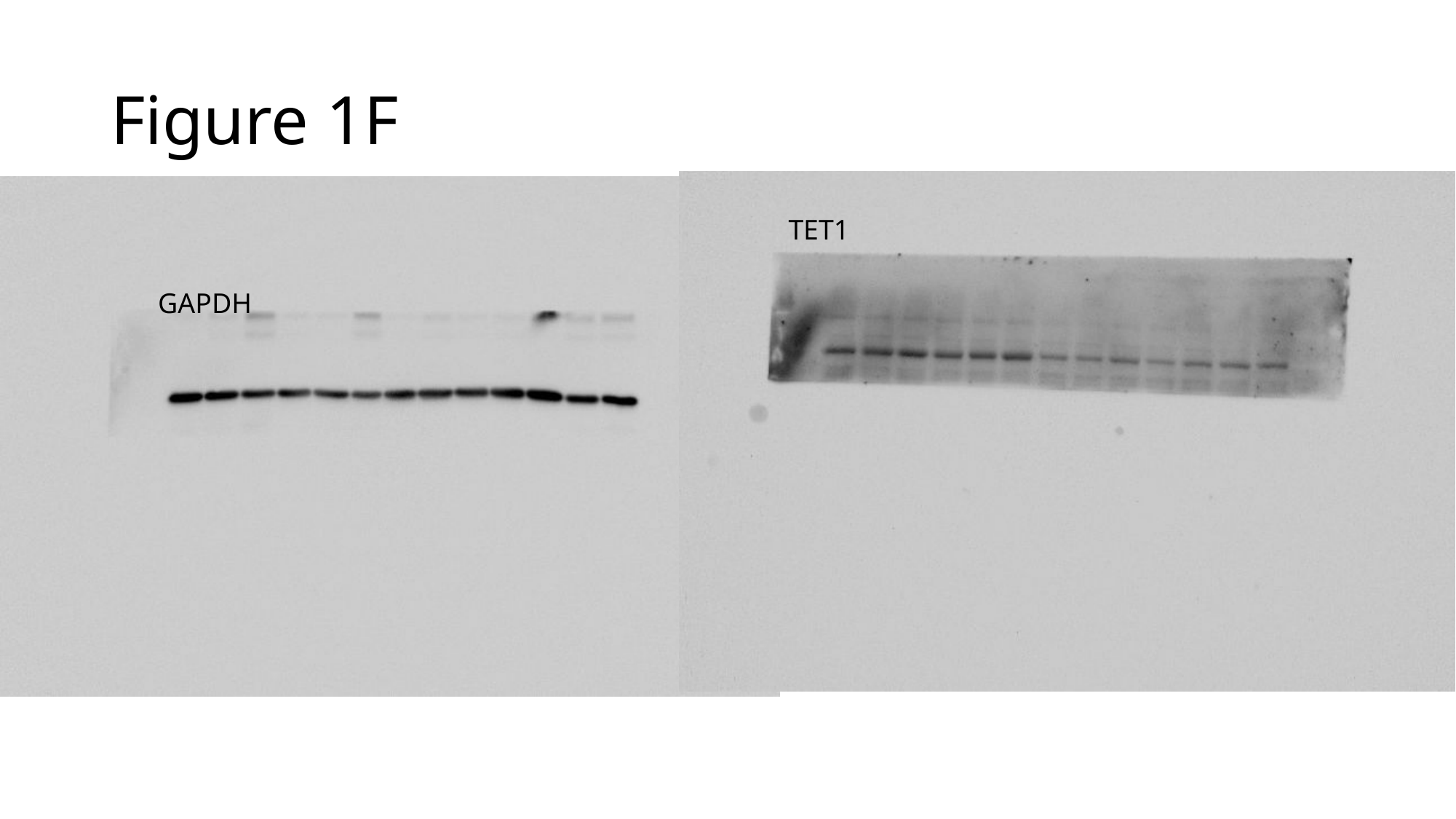

# Figure 1F
TET1
GAPDH

## Slide 2
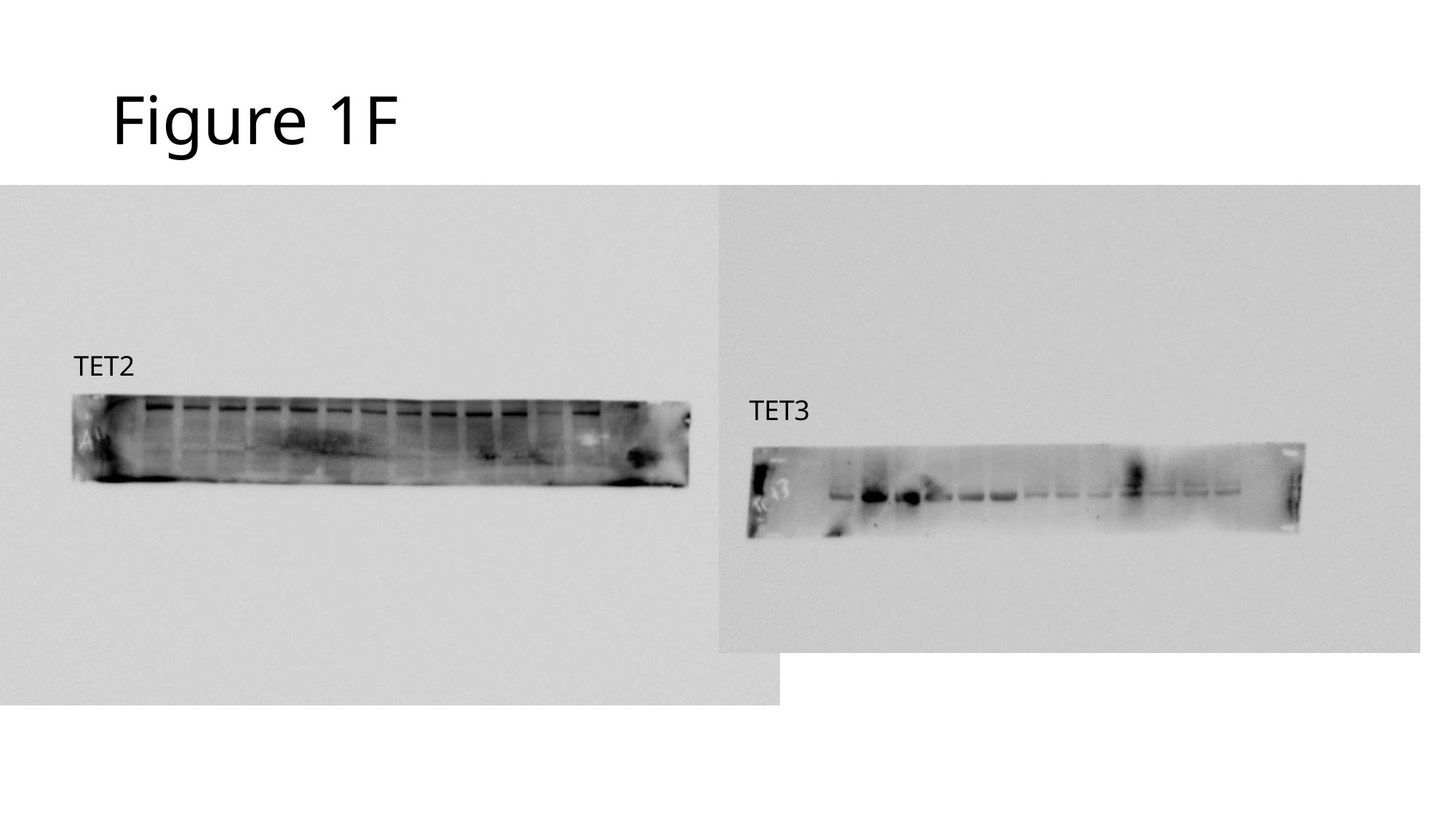

# Figure 1F
TET2
TET3

Supplement: Supplementary file 9 — Source data Fig. 1 [file 44321_2025_224_MOESM9_ESM.zip › Fig 1/Fig 1F.pptx]

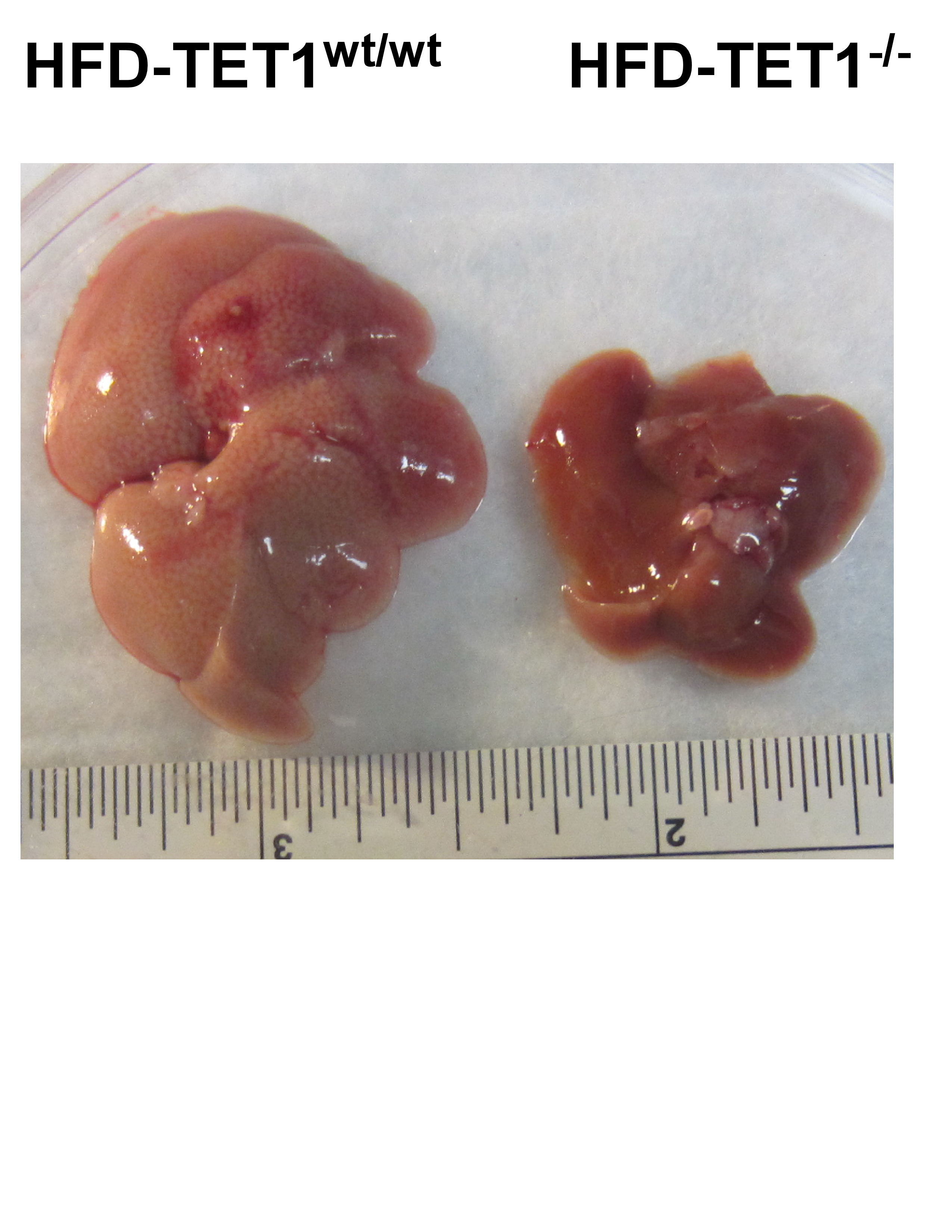

Supplement: Supplementary file 10 — Source data Fig. 2 [file 44321_2025_224_MOESM10_ESM.zip › Fig 2/Fig 2B.tif]

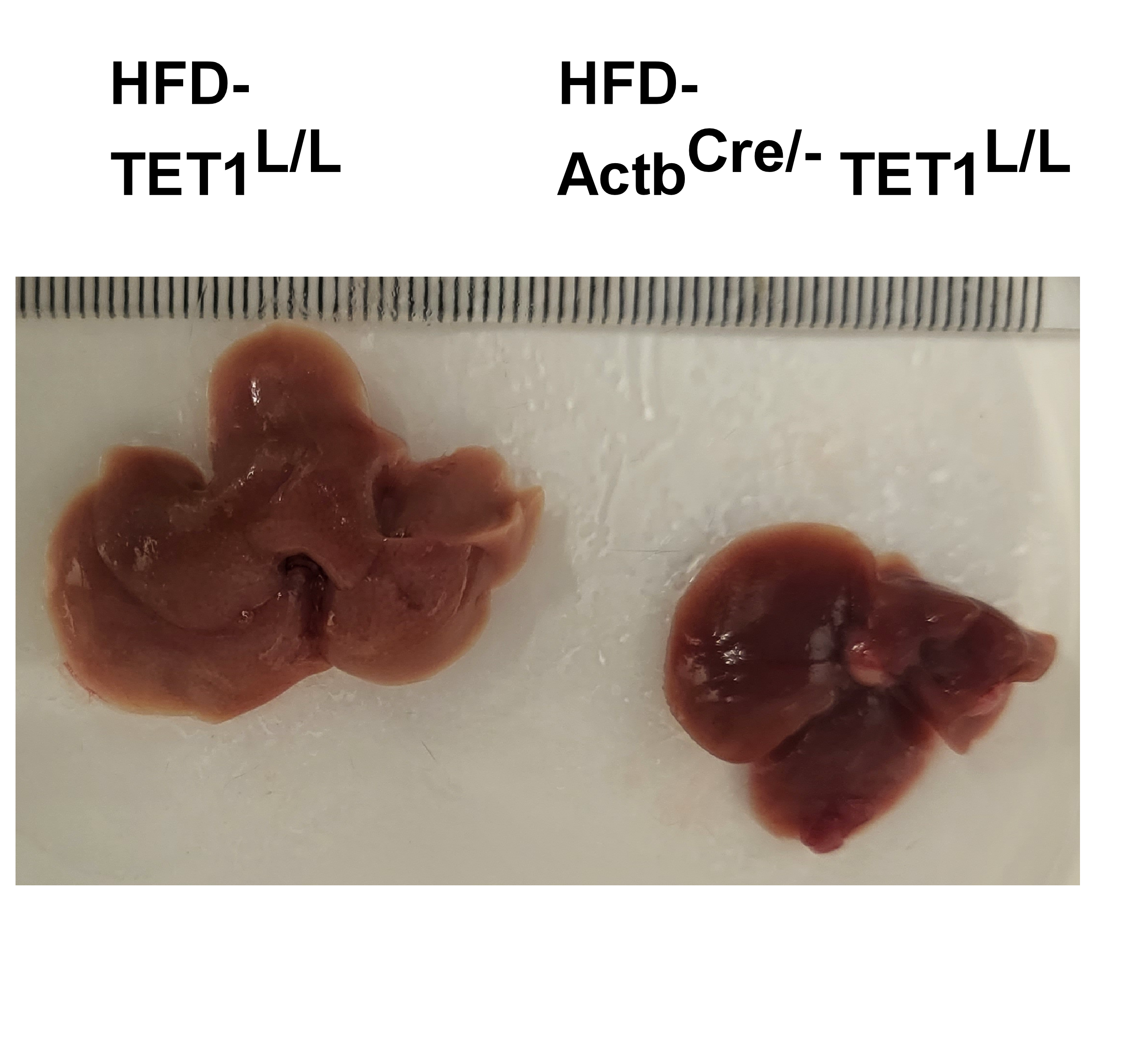

Supplement: Supplementary file 10 — Source data Fig. 2 [file 44321_2025_224_MOESM10_ESM.zip › Fig 2/Fig 2G.tif]

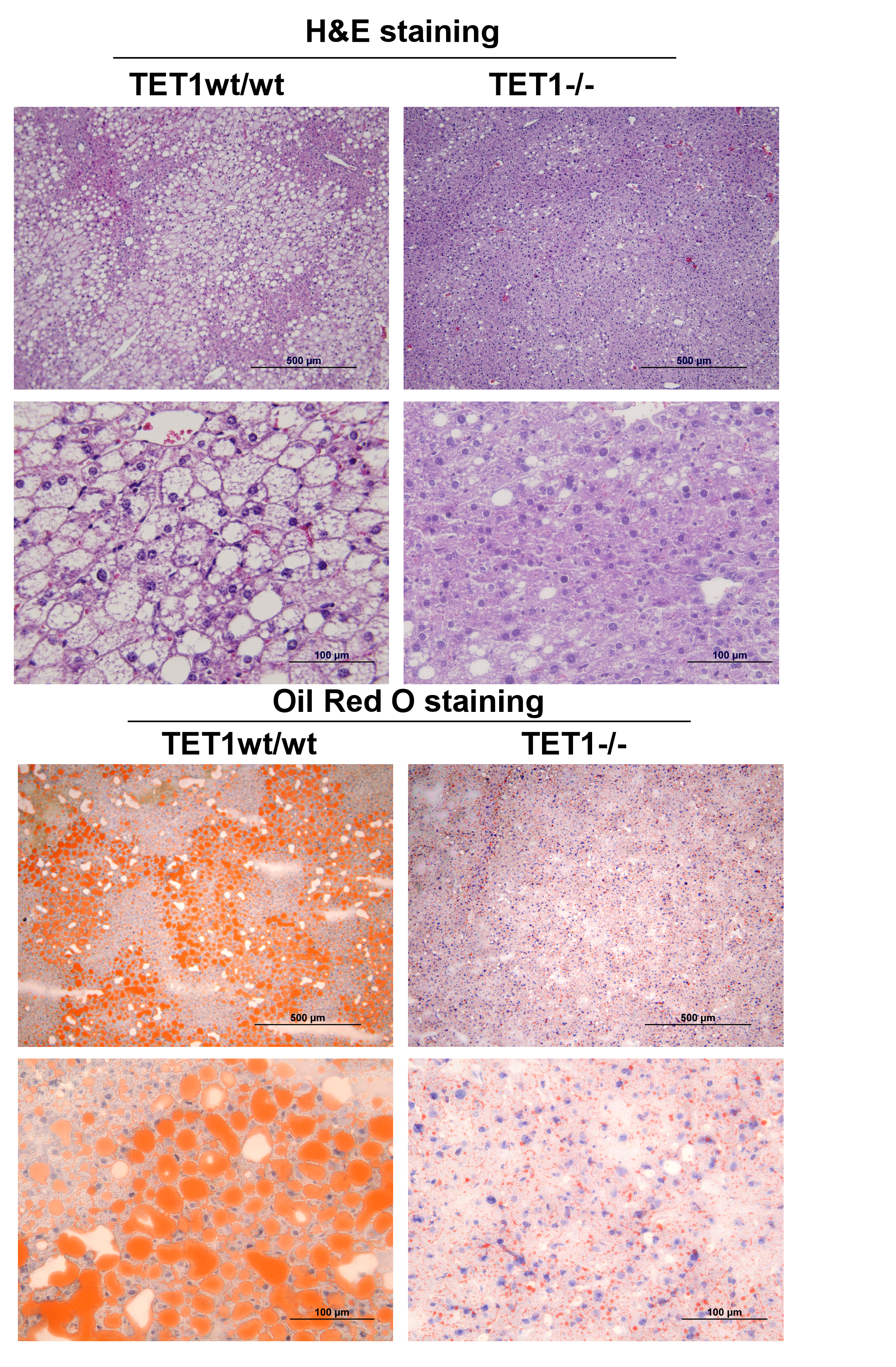

Supplement: Supplementary file 11 — Source data Fig. 3 [file 44321_2025_224_MOESM11_ESM.zip › Fig 3/Fig 3A.tif]

## Slide 1
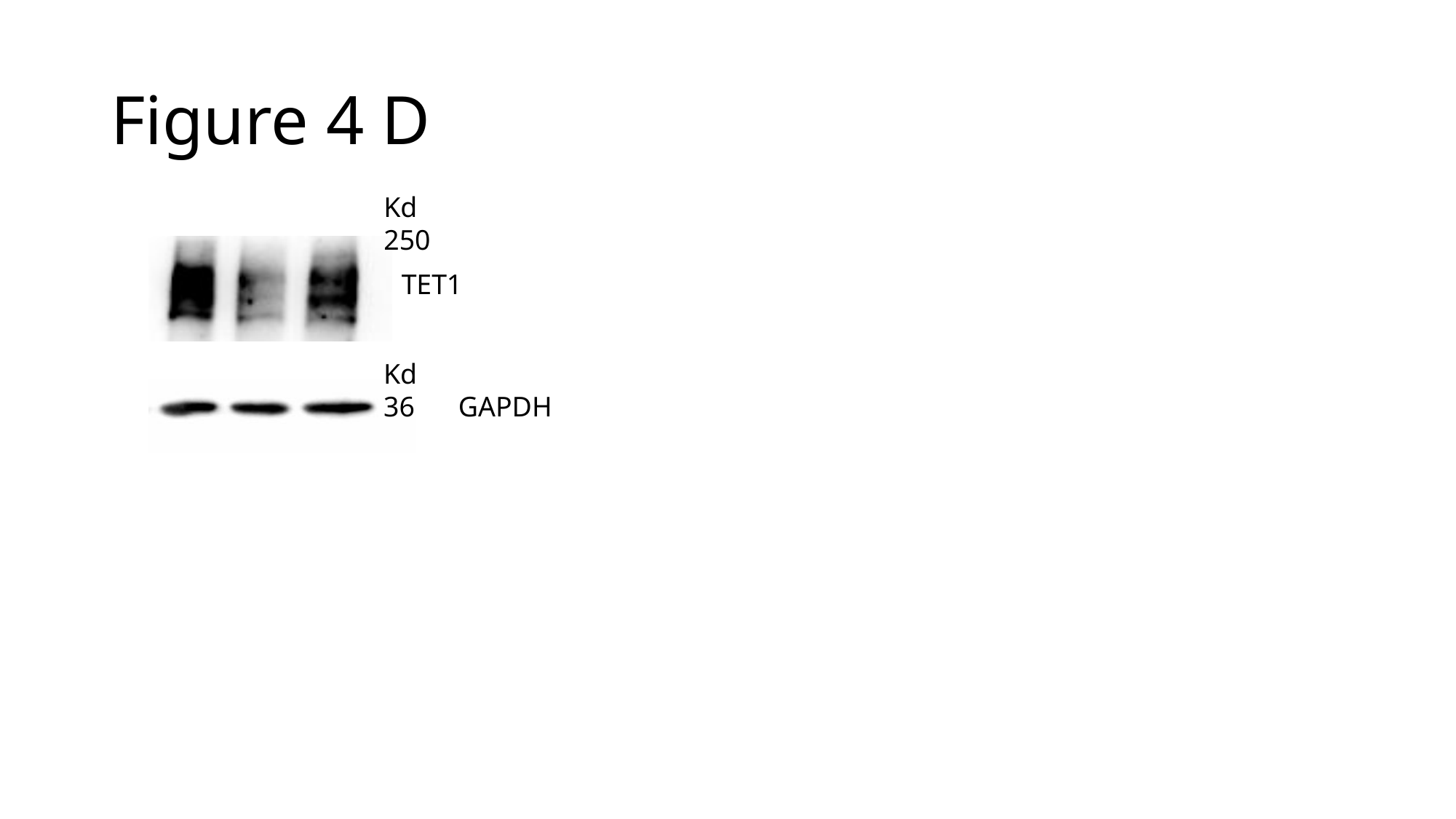

# Figure 4 D
Kd
250
TET1
Kd
36
GAPDH

Supplement: Supplementary file 12 — Source data Fig. 4 [file 44321_2025_224_MOESM12_ESM.zip › Fig 4/Fig 4D.pptx]

## Slide 1
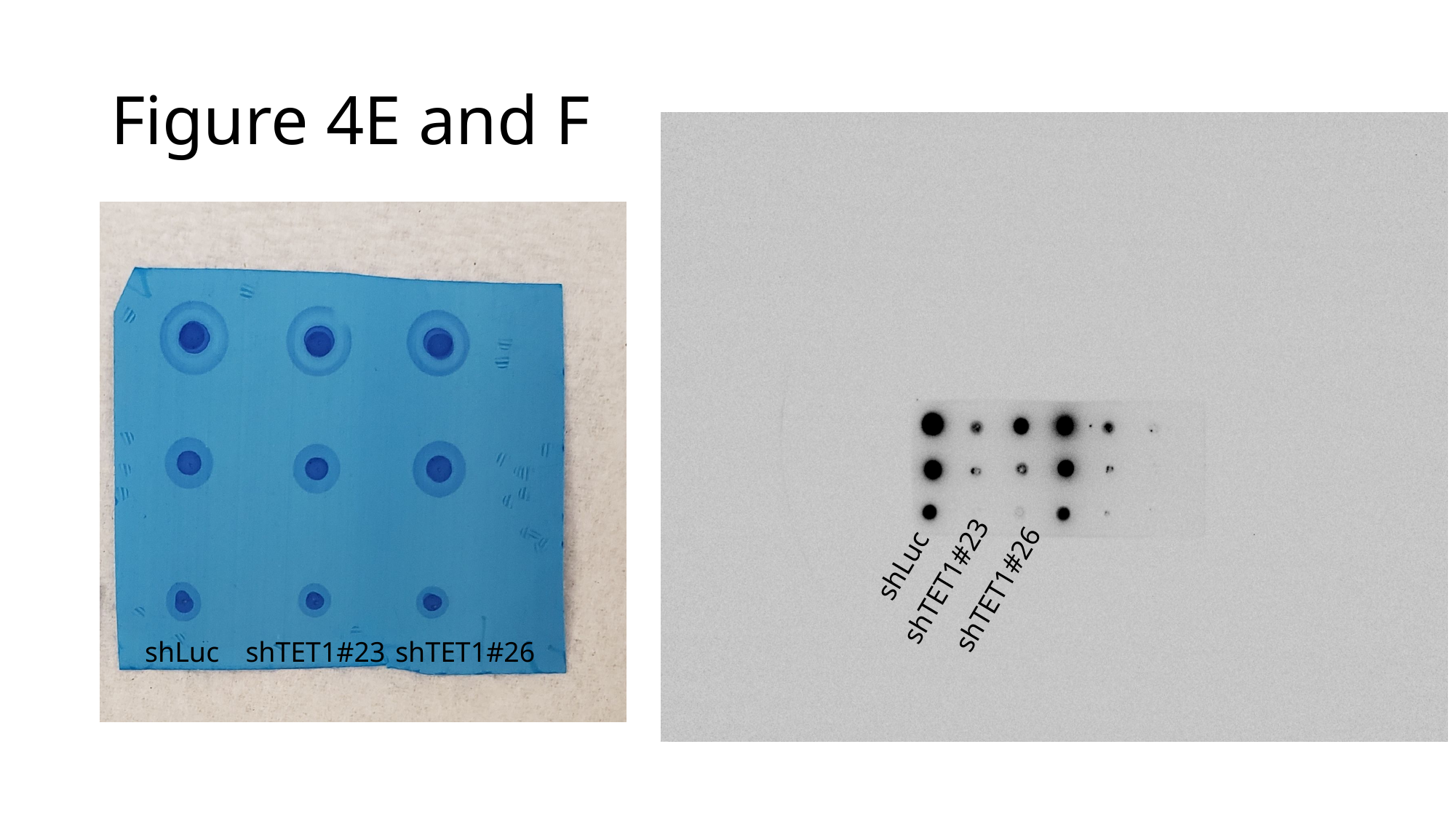

# Figure 4E and F
shLuc
shTET1#23
shTET1#26
shLuc
shTET1#23
shTET1#26

Supplement: Supplementary file 12 — Source data Fig. 4 [file 44321_2025_224_MOESM12_ESM.zip › Fig 4/Fig 4E and F.pptx]

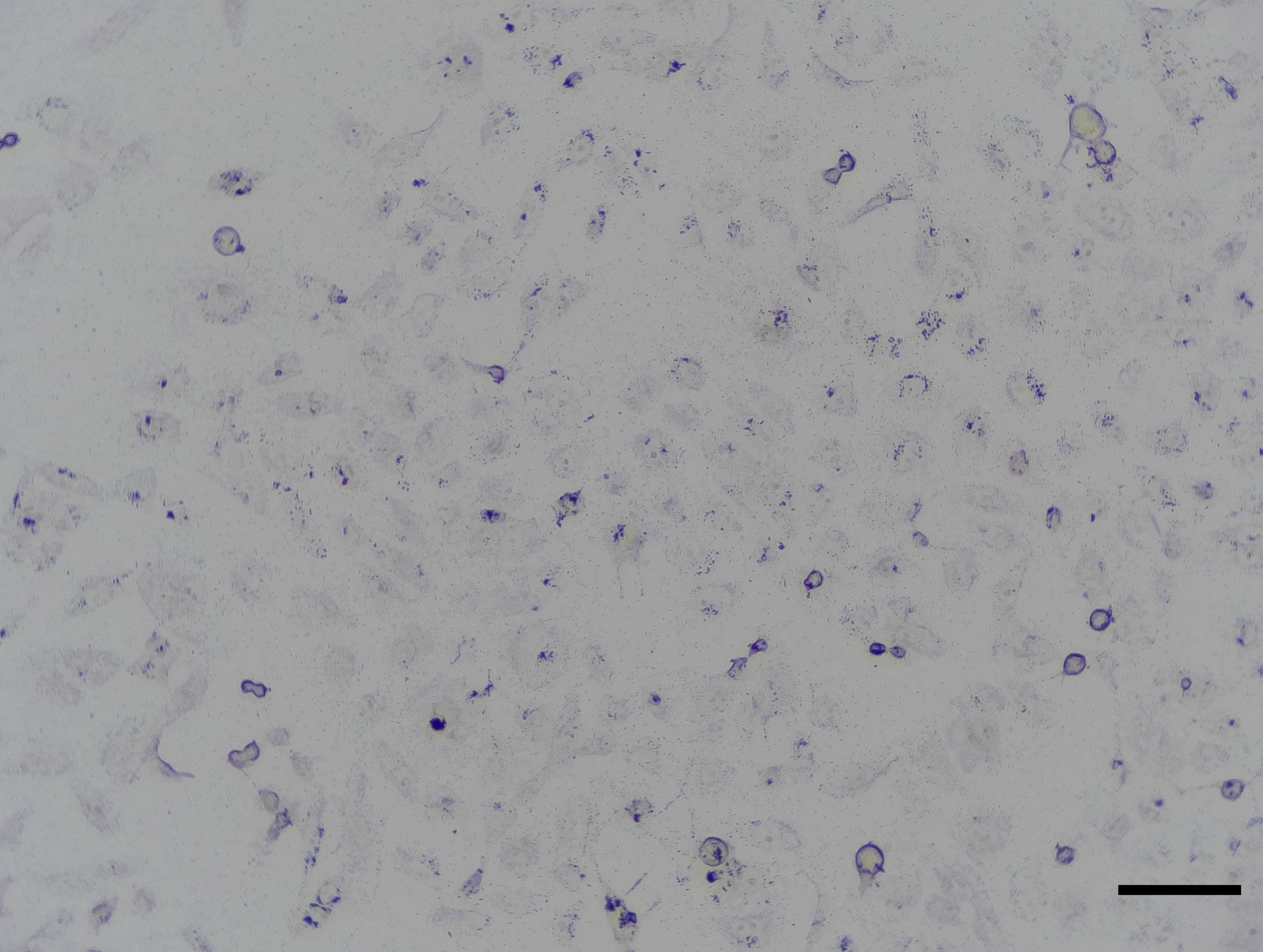

Supplement: Supplementary file 12 — Source data Fig. 4 [file 44321_2025_224_MOESM12_ESM.zip › Fig 4/Fig 4I/Ctrl-SC 10X SCALE.jpg]

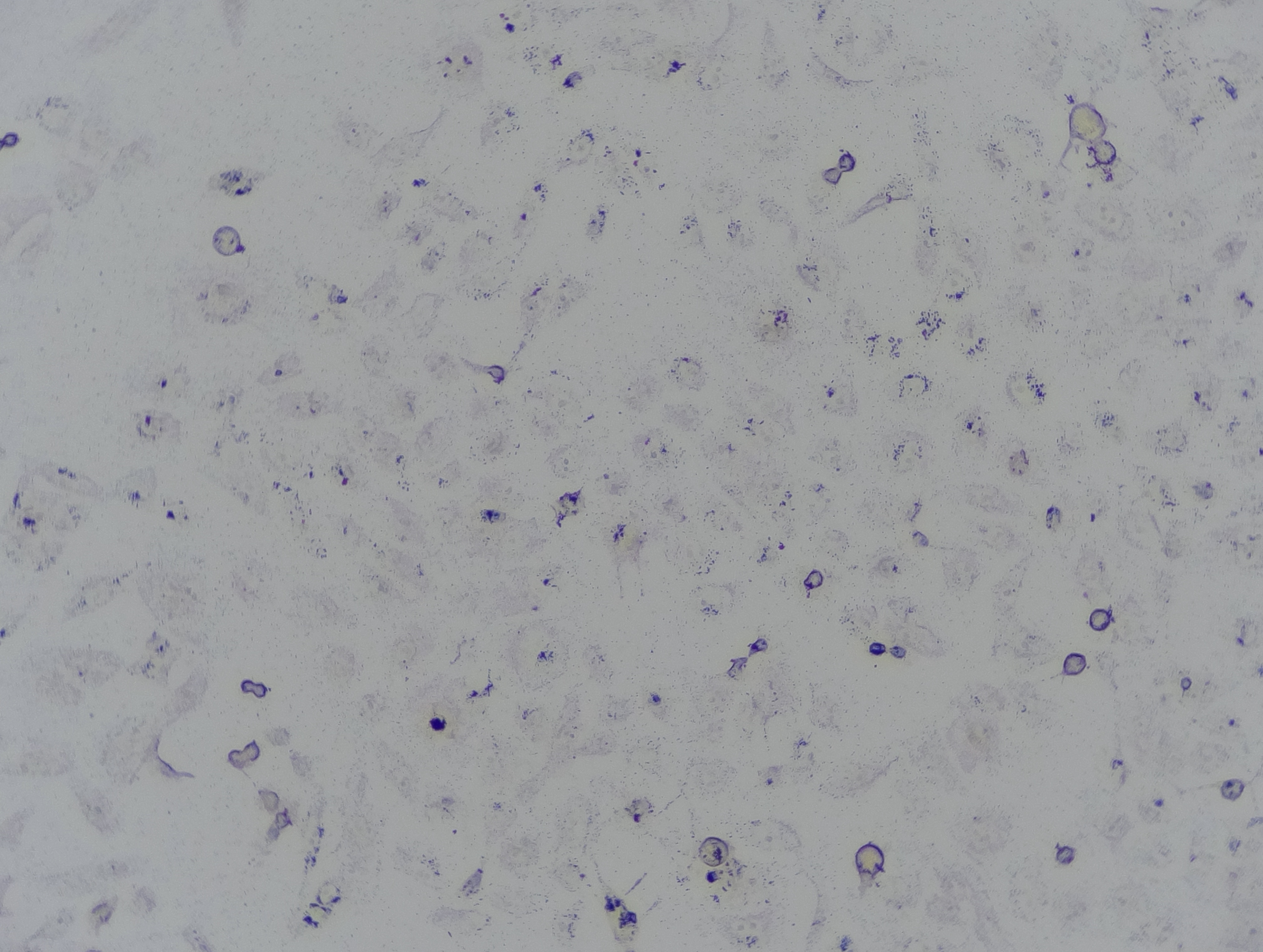

Supplement: Supplementary file 12 — Source data Fig. 4 [file 44321_2025_224_MOESM12_ESM.zip › Fig 4/Fig 4I/Ctrl-SC 10X.jpg]

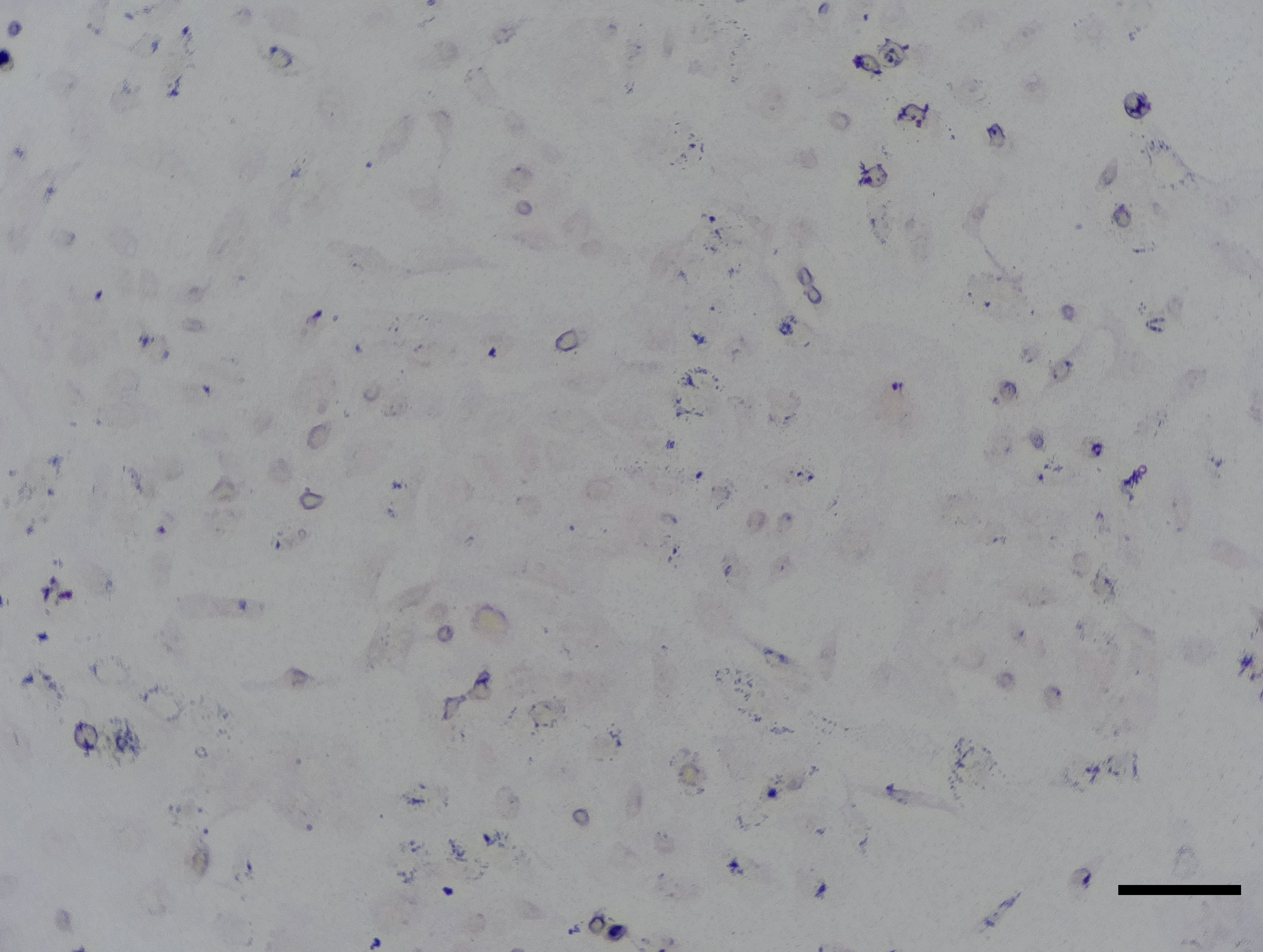

Supplement: Supplementary file 12 — Source data Fig. 4 [file 44321_2025_224_MOESM12_ESM.zip › Fig 4/Fig 4I/Ctrl-sh23-10x SCALE.jpg]

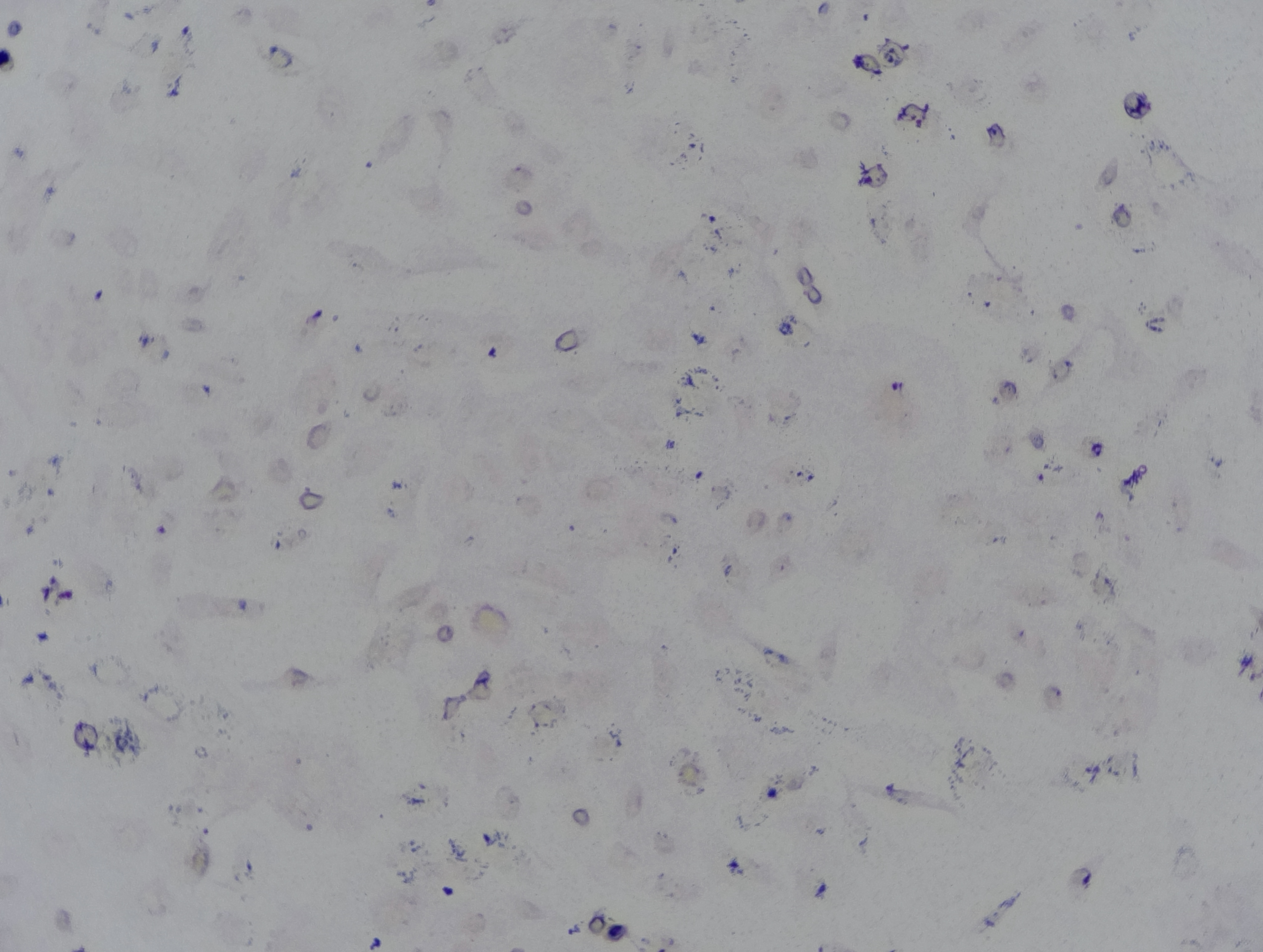

Supplement: Supplementary file 12 — Source data Fig. 4 [file 44321_2025_224_MOESM12_ESM.zip › Fig 4/Fig 4I/Ctrl-sh23-10x.jpg]

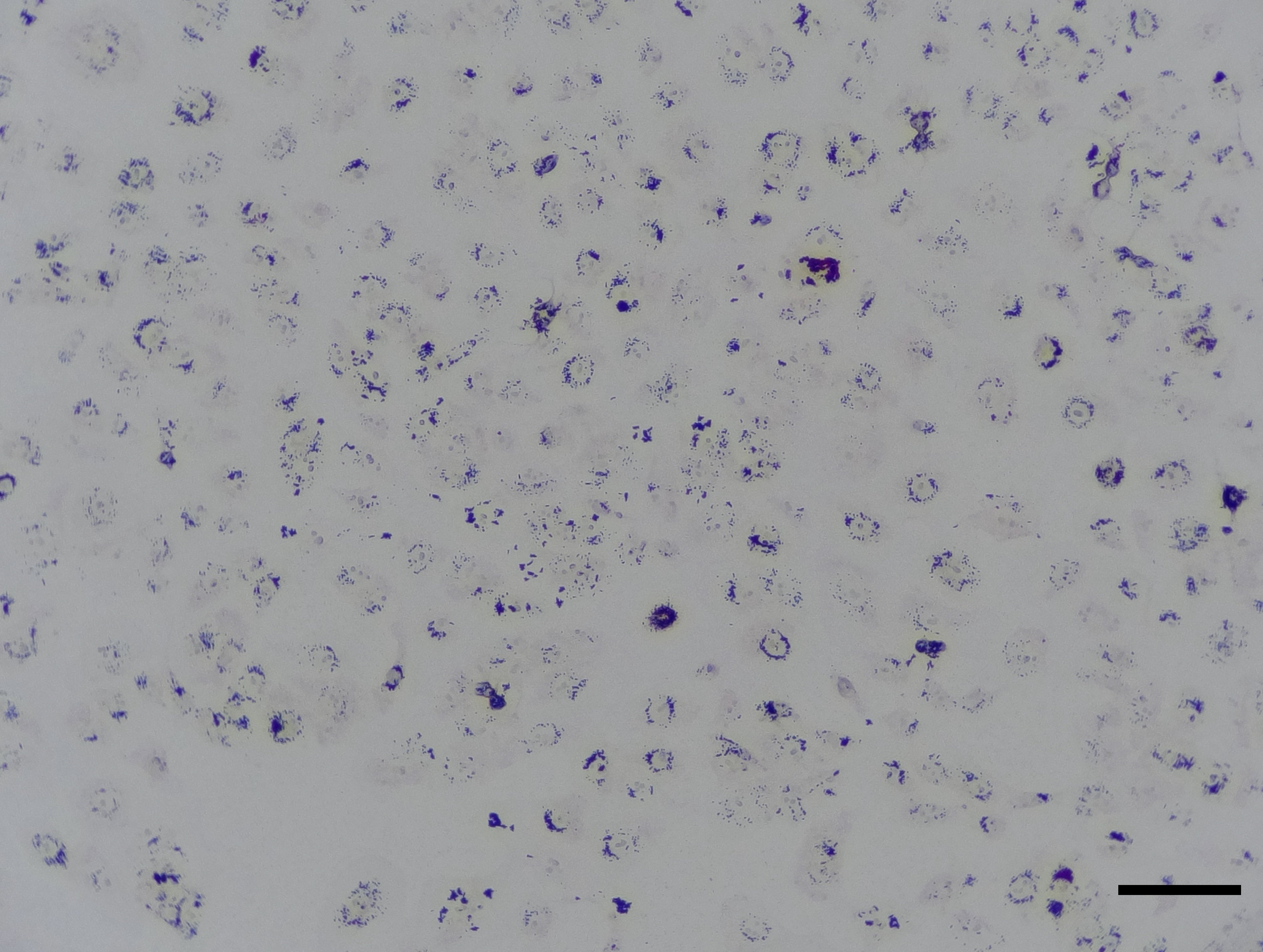

Supplement: Supplementary file 12 — Source data Fig. 4 [file 44321_2025_224_MOESM12_ESM.zip › Fig 4/Fig 4I/Ctrl-Sh26-10X SCALE.jpg]

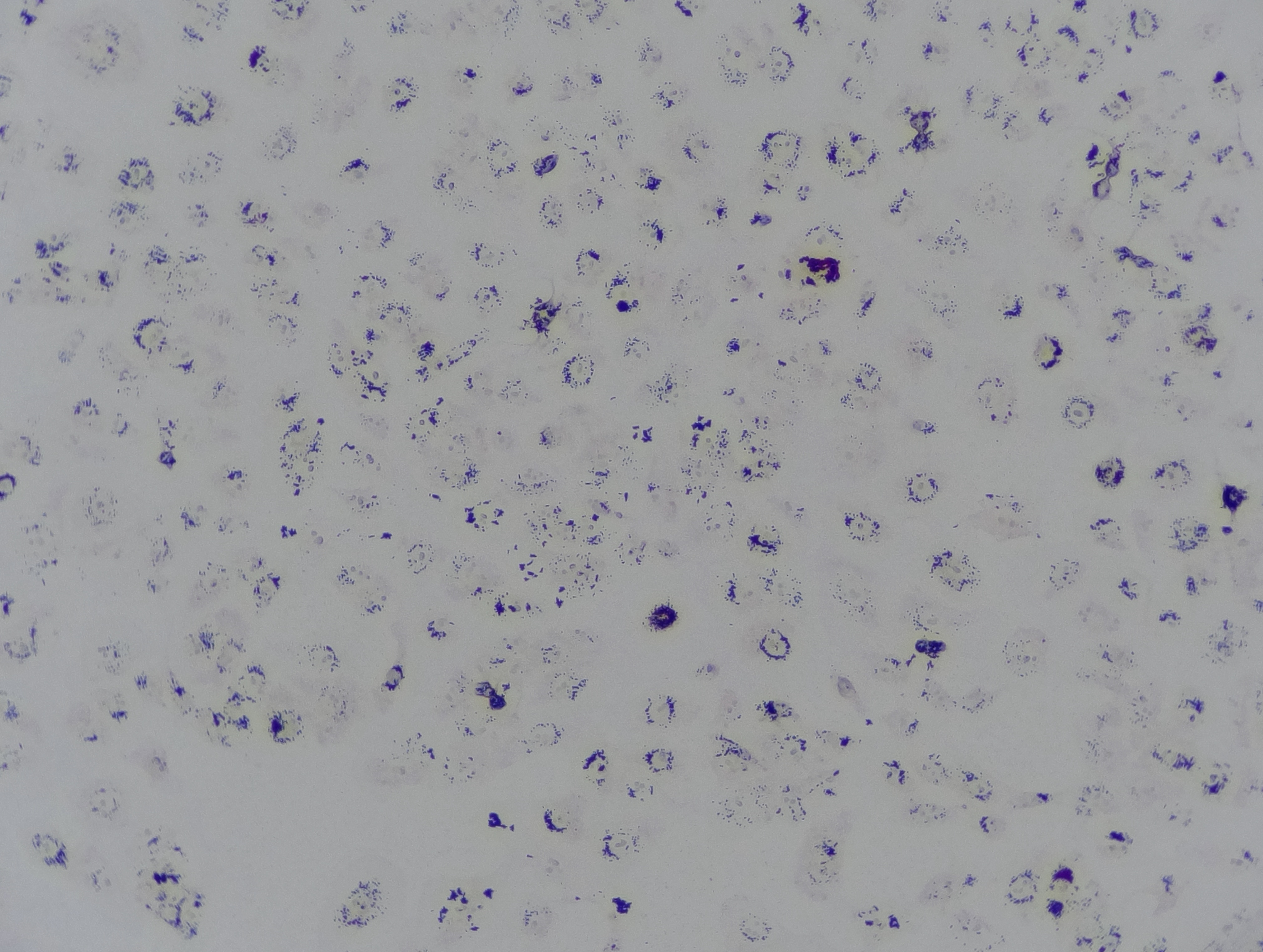

Supplement: Supplementary file 12 — Source data Fig. 4 [file 44321_2025_224_MOESM12_ESM.zip › Fig 4/Fig 4I/Ctrl-Sh26-10X.jpg]

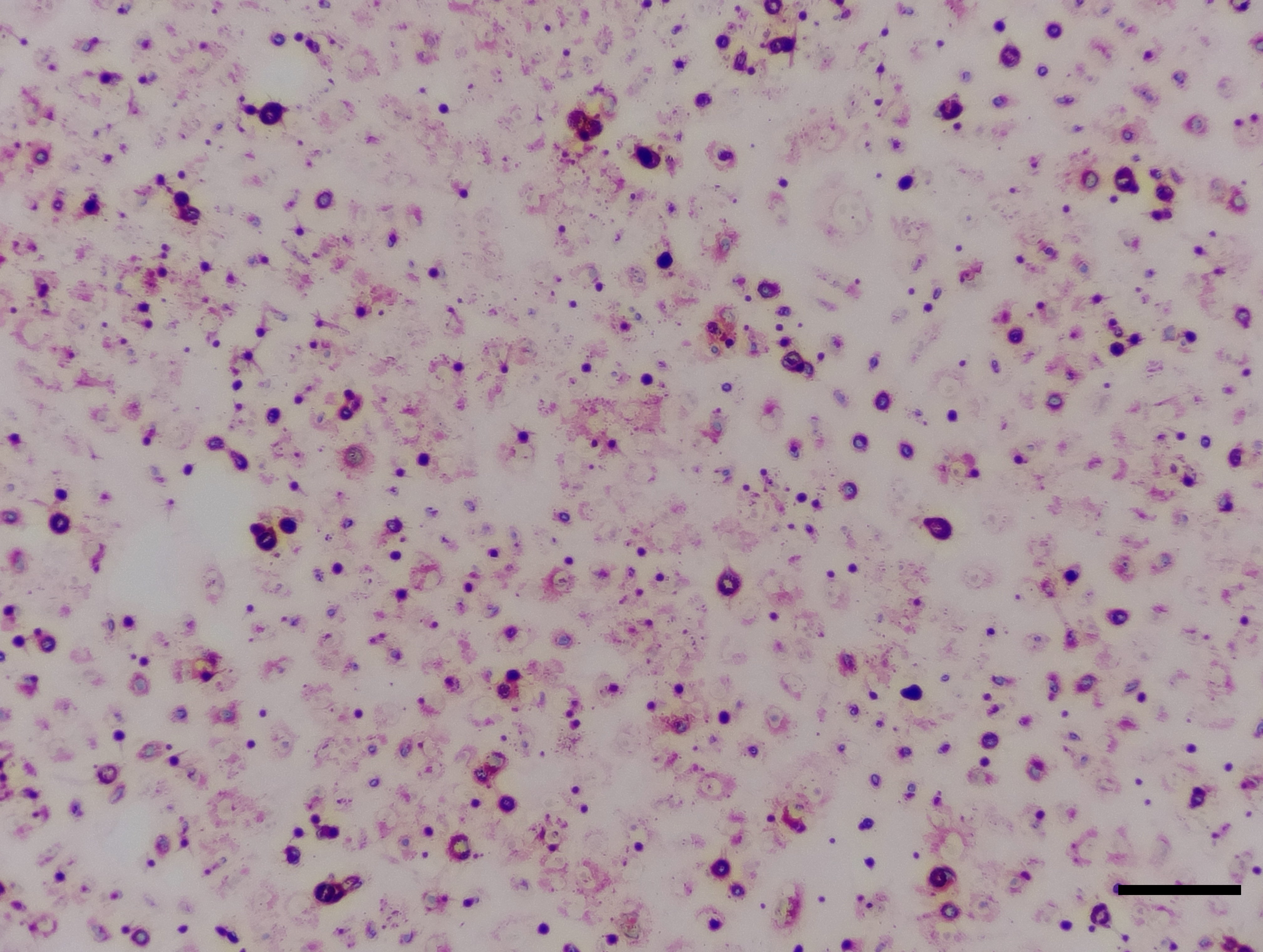

Supplement: Supplementary file 12 — Source data Fig. 4 [file 44321_2025_224_MOESM12_ESM.zip › Fig 4/Fig 4I/OA-sh23-10x SCALE.jpg]

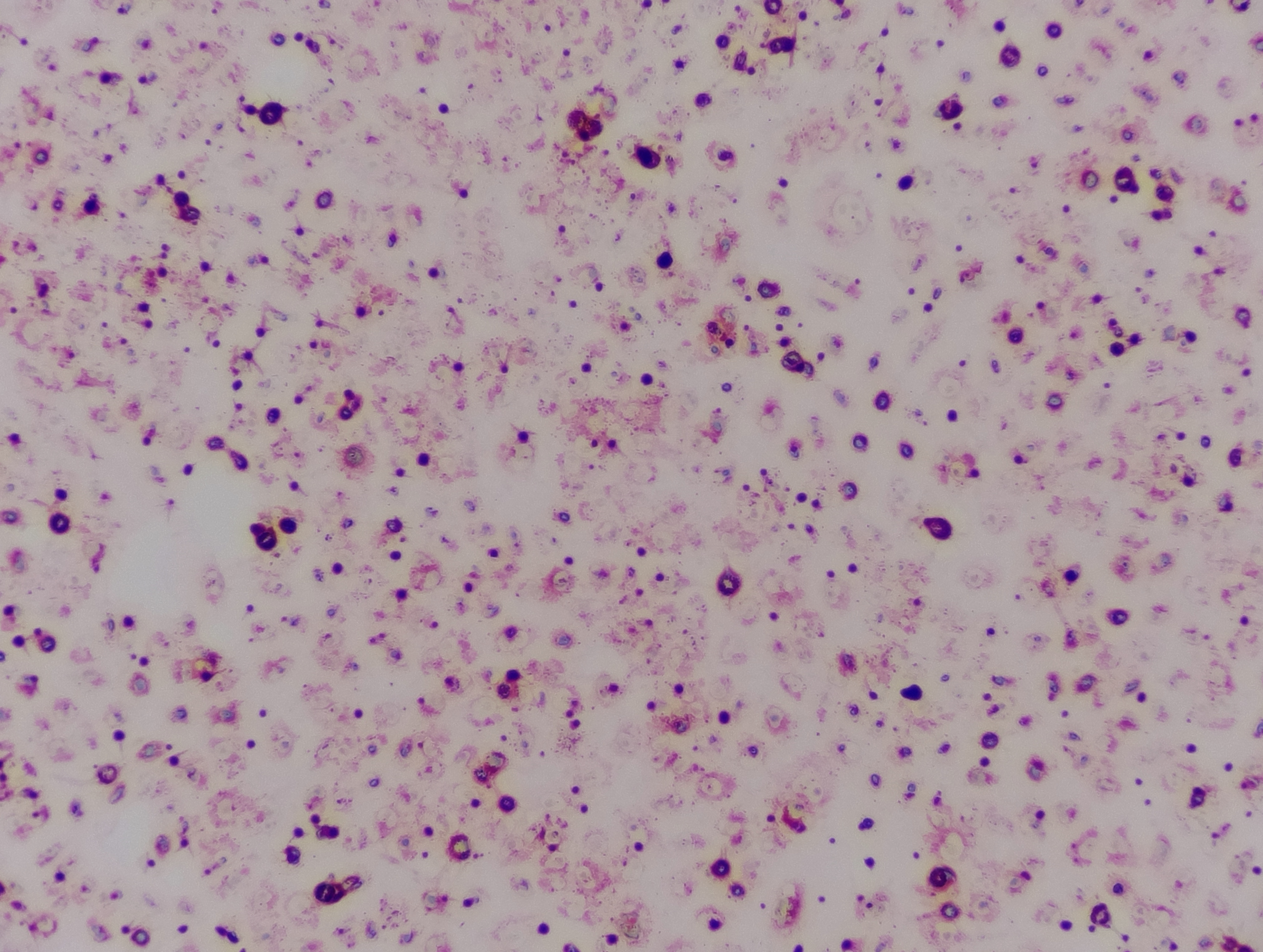

Supplement: Supplementary file 12 — Source data Fig. 4 [file 44321_2025_224_MOESM12_ESM.zip › Fig 4/Fig 4I/OA-sh23-10x.jpg]

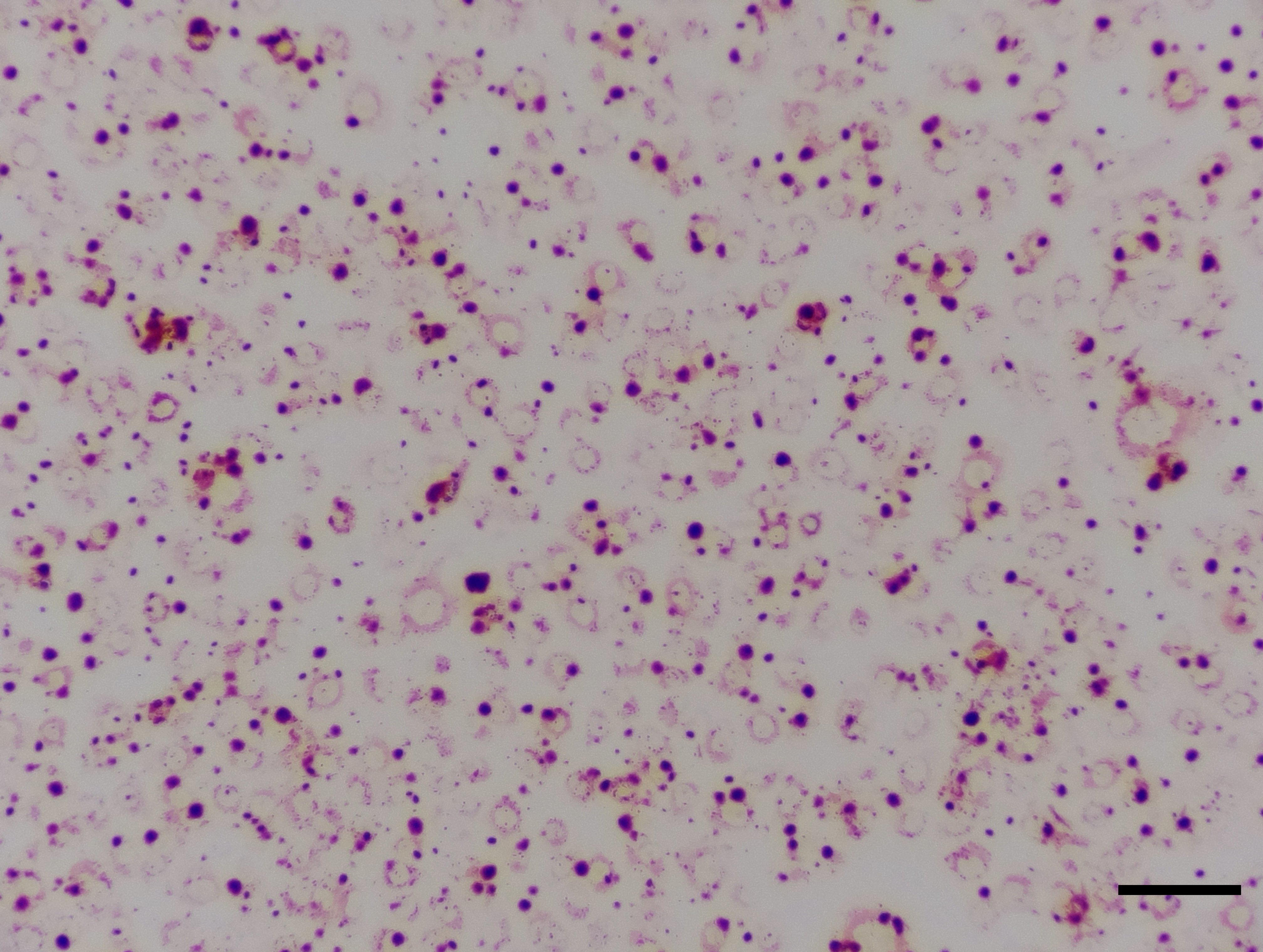

Supplement: Supplementary file 12 — Source data Fig. 4 [file 44321_2025_224_MOESM12_ESM.zip › Fig 4/Fig 4I/OA-sh26-10x SCALE.jpg]

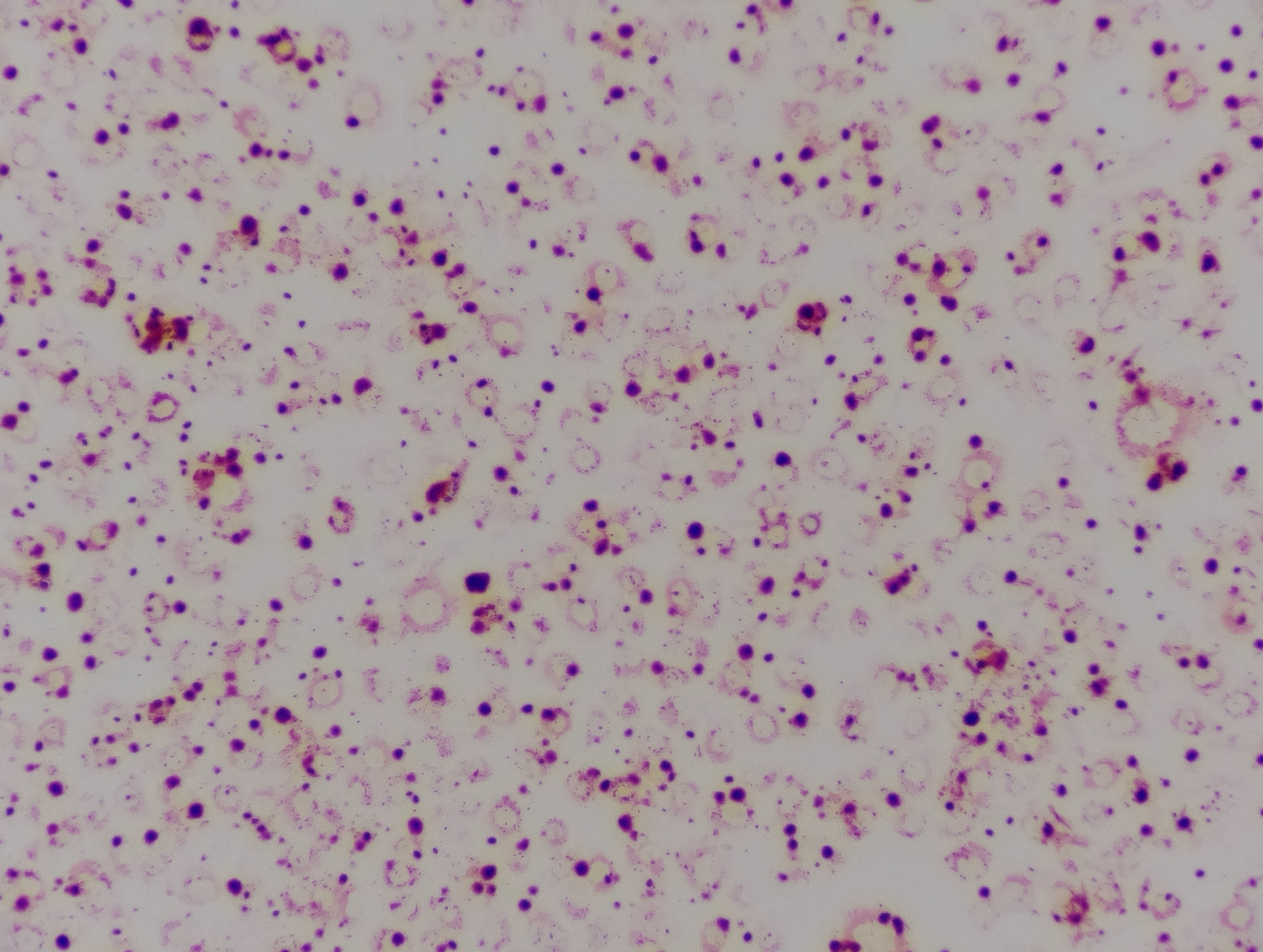

Supplement: Supplementary file 12 — Source data Fig. 4 [file 44321_2025_224_MOESM12_ESM.zip › Fig 4/Fig 4I/OA-sh26-10x.jpg]

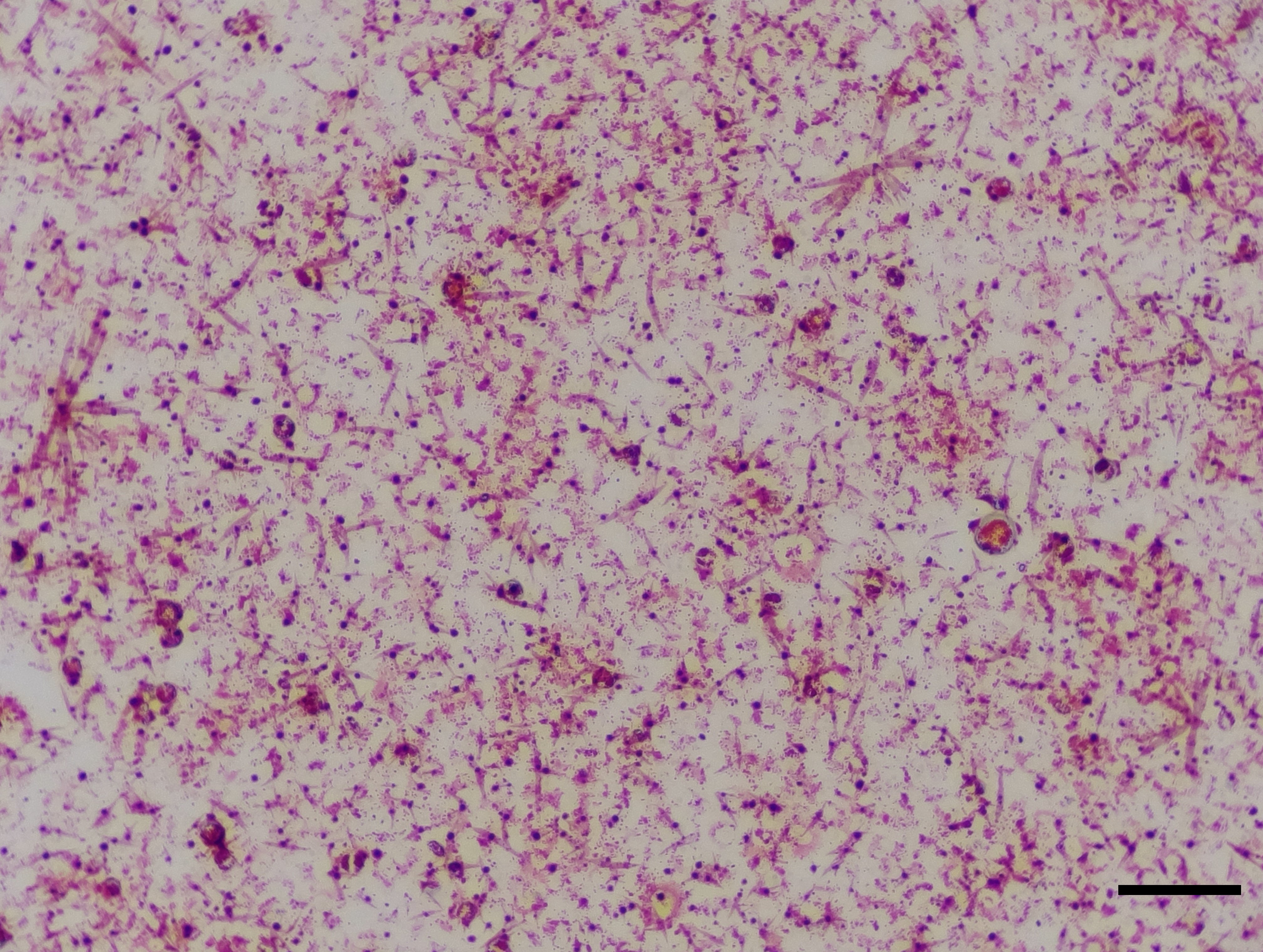

Supplement: Supplementary file 12 — Source data Fig. 4 [file 44321_2025_224_MOESM12_ESM.zip › Fig 4/Fig 4I/SC-OA 10X SCAL.jpg]

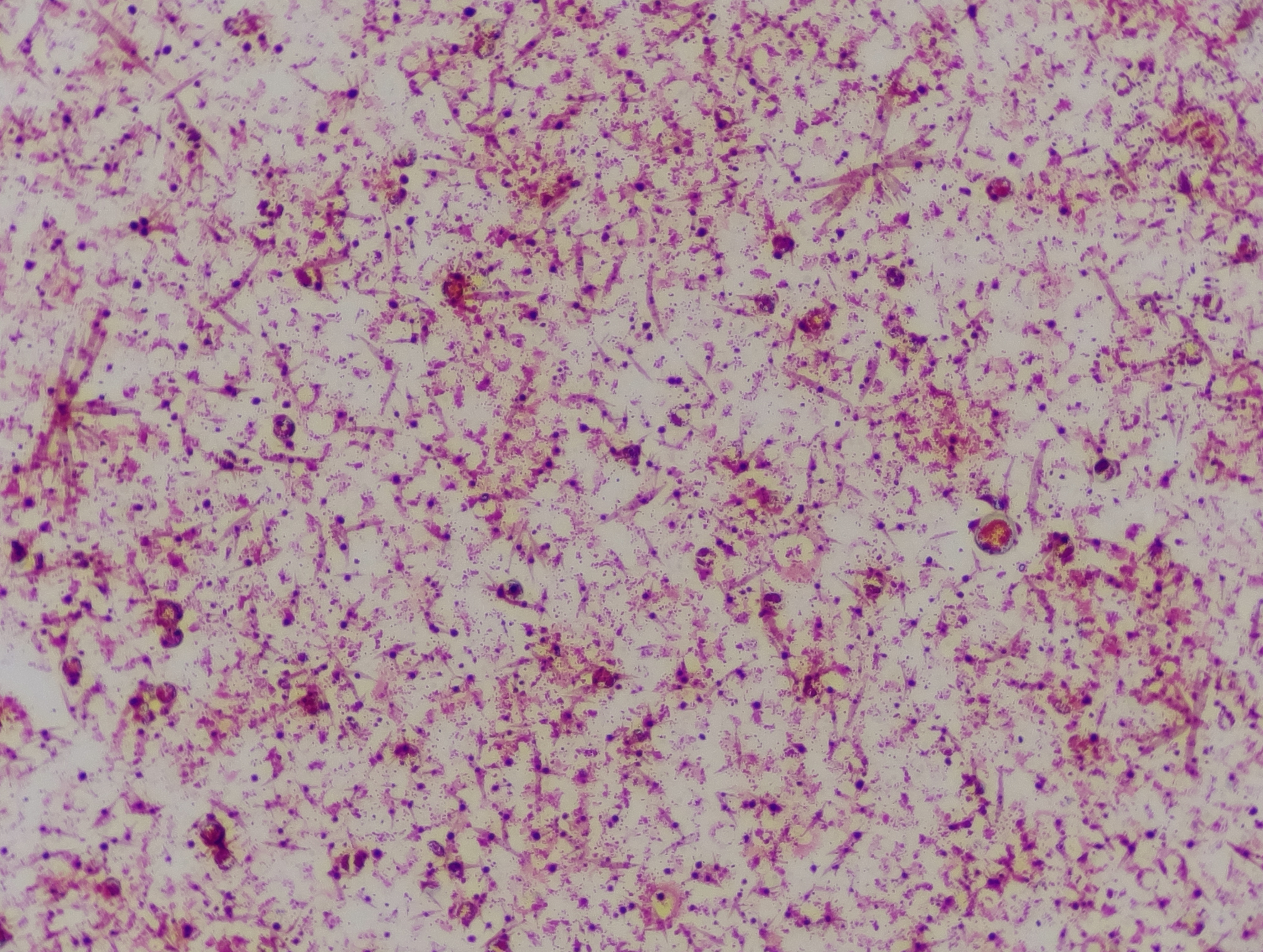

Supplement: Supplementary file 12 — Source data Fig. 4 [file 44321_2025_224_MOESM12_ESM.zip › Fig 4/Fig 4I/SC-OA 10X.jpg]

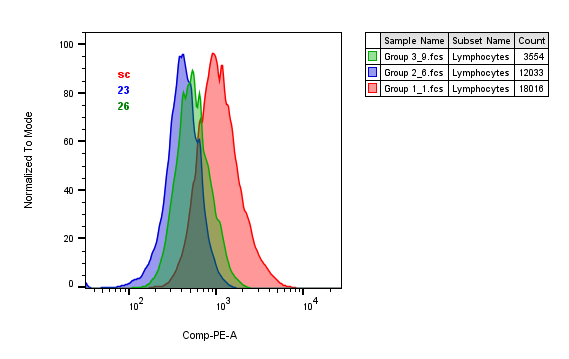

Supplement: Supplementary file 12 — Source data Fig. 4 [file 44321_2025_224_MOESM12_ESM.zip › Fig 4/fig 4K/24-Apr-2023-Layout.tiff]

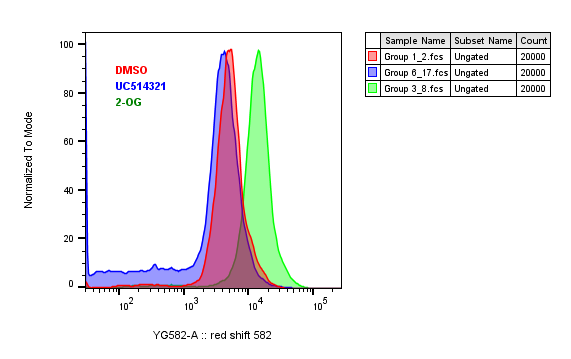

Supplement: Supplementary file 12 — Source data Fig. 4 [file 44321_2025_224_MOESM12_ESM.zip › Fig 4/fig 4M/16-May-2023-Layout.tiff]

## Slide 1
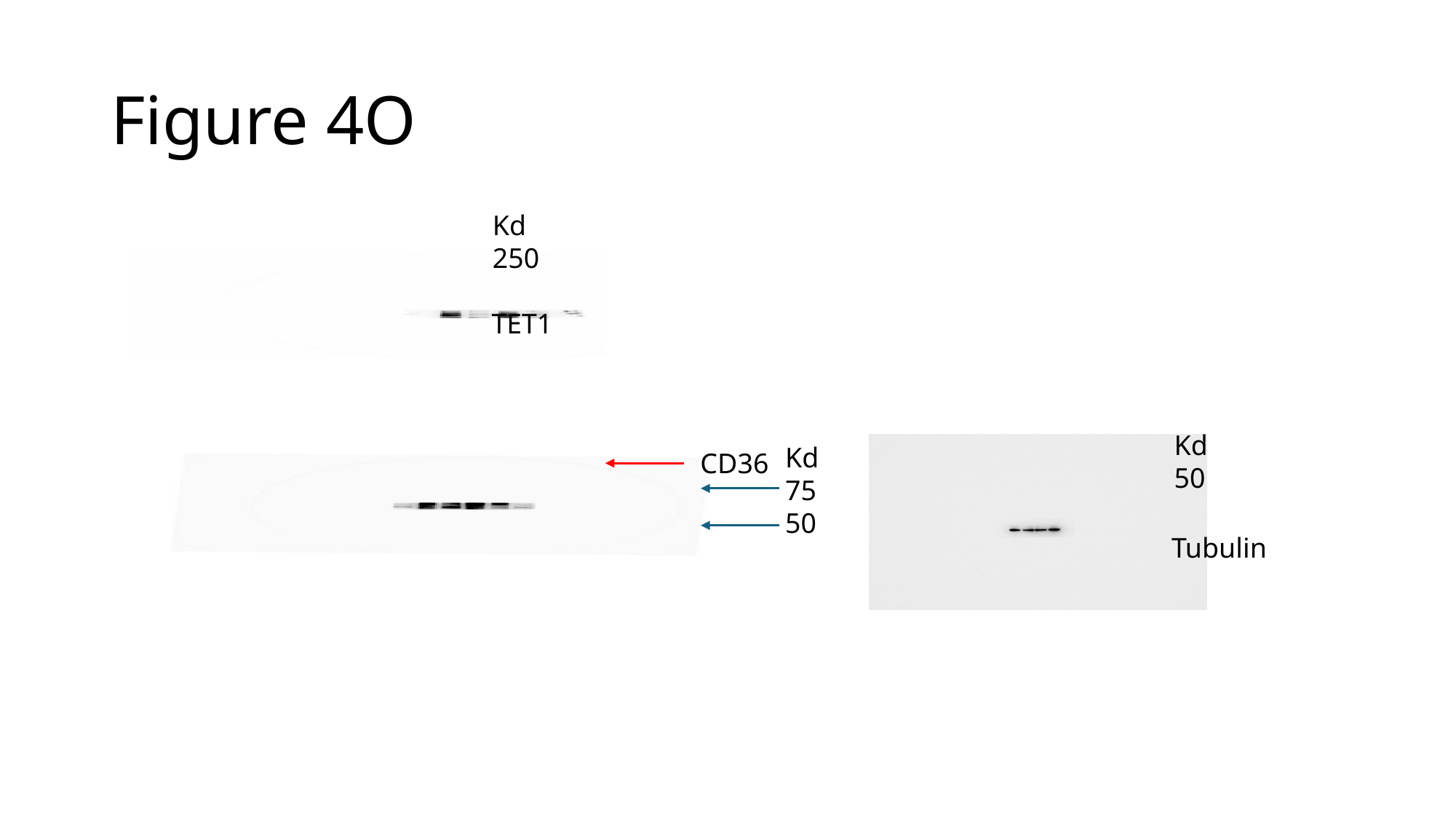

# Figure 4O
Kd
250
TET1
Kd
50
Kd
75
50
CD36
Tubulin

Supplement: Supplementary file 12 — Source data Fig. 4 [file 44321_2025_224_MOESM12_ESM.zip › Fig 4/Fig 4O.pptx]

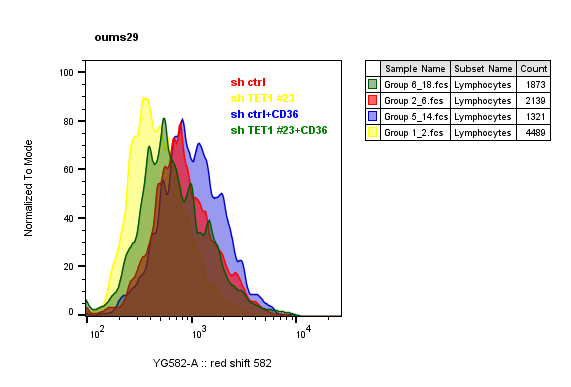

Supplement: Supplementary file 12 — Source data Fig. 4 [file 44321_2025_224_MOESM12_ESM.zip › Fig 4/fig 4P/14-Sep-2023-Layout.tiff]

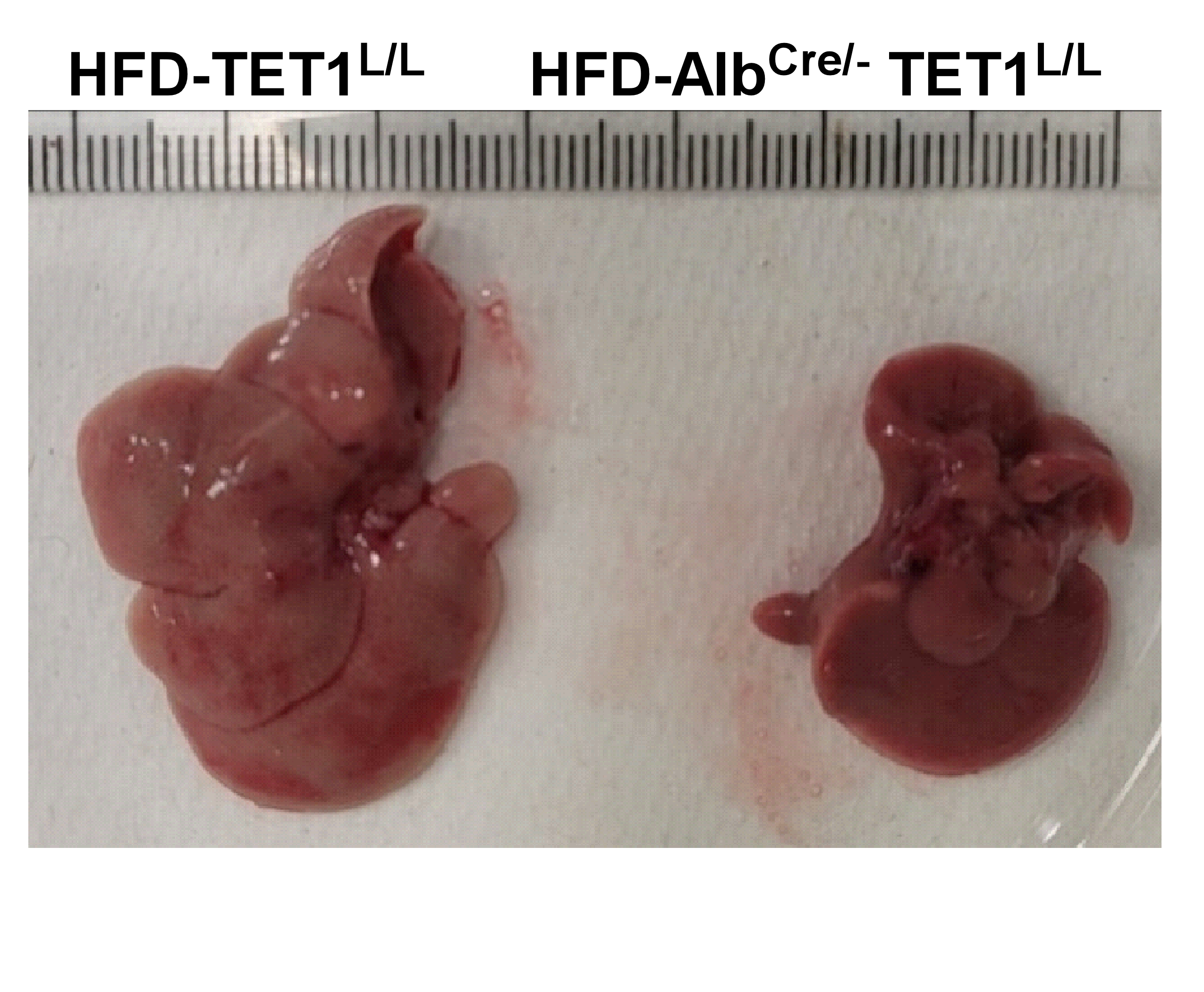

Supplement: Supplementary file 14 — Source data Fig. 6 [file 44321_2025_224_MOESM14_ESM.zip › Fig 6/Fig 6B.tif]

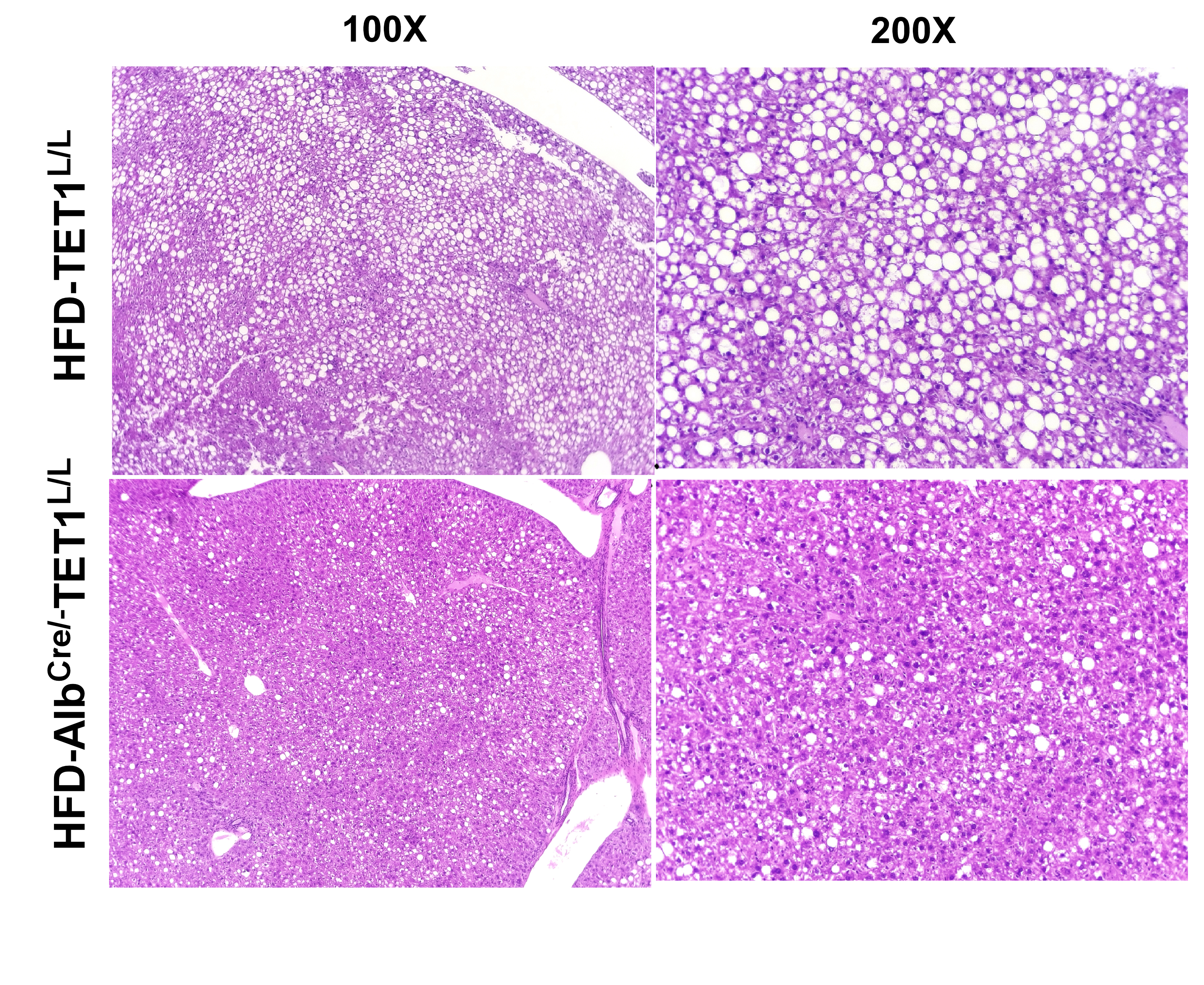

Supplement: Supplementary file 14 — Source data Fig. 6 [file 44321_2025_224_MOESM14_ESM.zip › Fig 6/Fig 6F.tif]

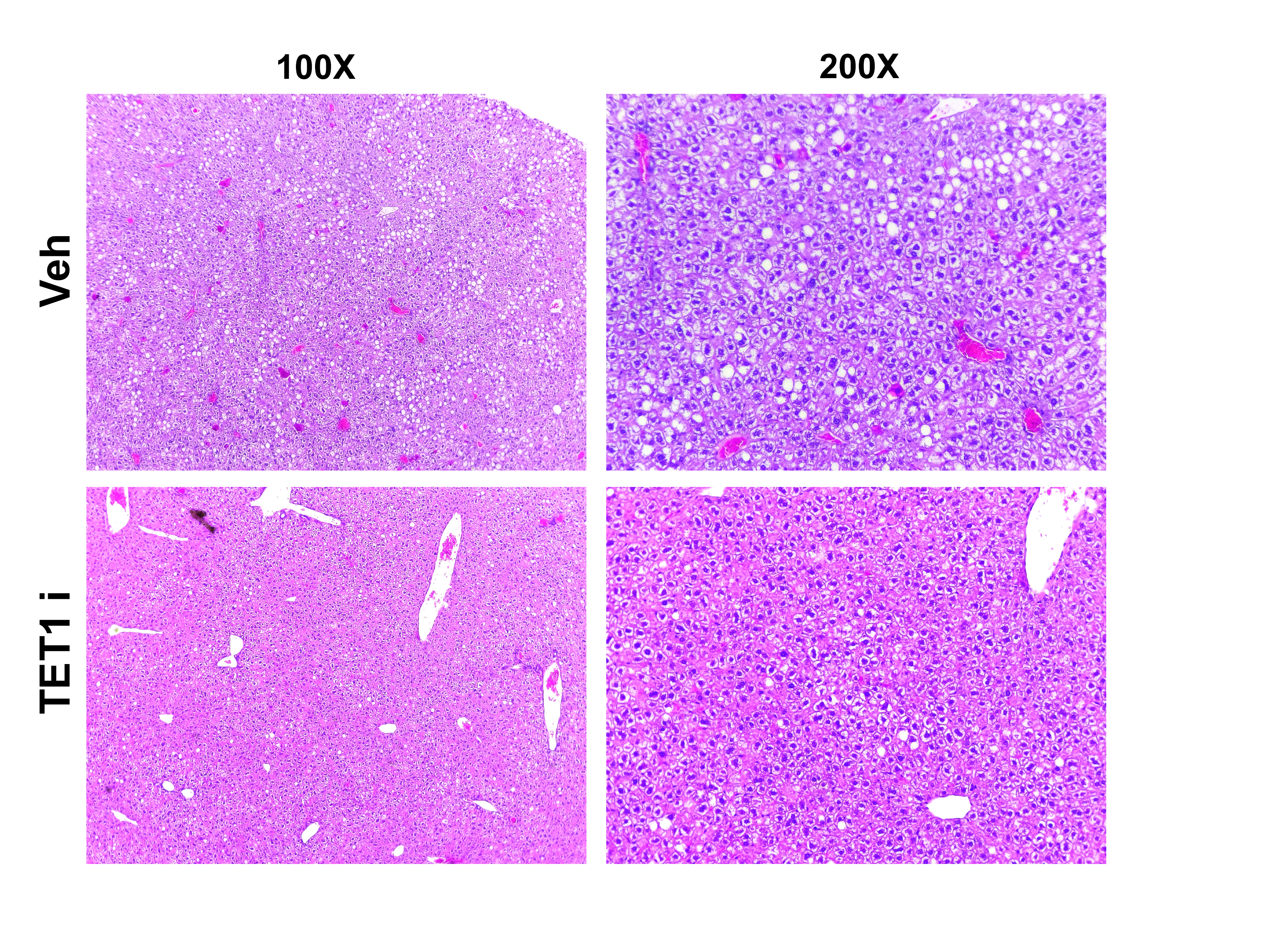

Supplement: Supplementary file 15 — Source data Fig. 7 [file 44321_2025_224_MOESM15_ESM.zip › Fig 7/Fig 7G.tif]

## Slide 1
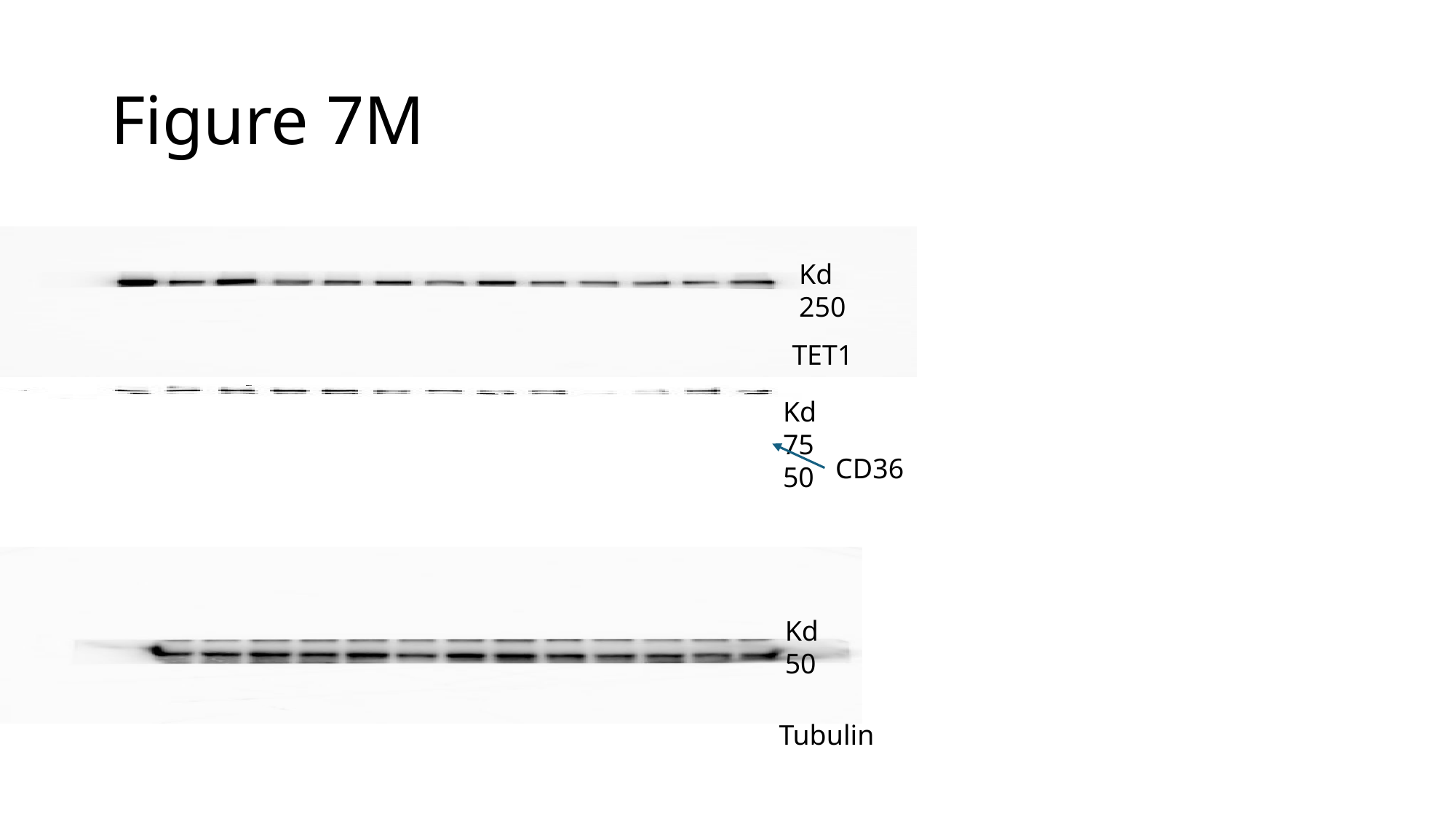

# Figure 7M
Kd
250
TET1
Kd
75
50
CD36
Kd
50
Tubulin

Supplement: Supplementary file 15 — Source data Fig. 7 [file 44321_2025_224_MOESM15_ESM.zip › Fig 7/Fig 7M.pptx]
